# DNA damage-induced paraspeckle formation enhances DNA repair and tumor radioresistance by recruiting ribosomal protein P0

**DOI:** 10.1038/s41419-022-05092-1

**Published:** 2022-08-16

**Authors:** Yun-Long Wang, Wan-Wen Zhao, Shao-Mei Bai, Yan Ma, Xin-Ke Yin, Li-Li Feng, Guang-Dong Zeng, Fang Wang, Wei-Xing Feng, Jian Zheng, Ying-Nai Wang, Bing Zeng, Quentin Liu, Mien-Chie Hung, Xiang-Bo Wan

**Affiliations:** 1 Guangdong Institute of Gastroenterology, Guangdong Provincial Key Laboratory of Colorectal and Pelvic Floor Diseases, the Sixth Affiliated Hospital, Sun Yat-sen University, Guangzhou, Guangdong 510655 People’s Republic of China; 2 Department of Radiation Oncology, the Sixth Affiliated Hospital, Sun Yat-sen University, Guangzhou, Guangdong 510655 People’s Republic of China; 3 Department of Molecular and Cellular Oncology, The University of Texas MD Anderson Cancer Center, Houston, TX 77030 USA; 4 Institute of Cancer Stem Cell, Dalian Medical University, Dalian, Liaoning 116044 People’s Republic of China; 5 State Key Laboratory of Oncology in South China, Cancer Center, Sun Yat-sen University, Guangzhou, Guangdong 510060 People’s Republic of China; 6 Graduate Institute of Biomedical Sciences and Research Centers for Cancer Biology and Molecular Medicine, China Medical University, Taichung, 404 Taiwan; 7 Department of Biotechnology, Asia University, Taichung, 413 Taiwan

**Keywords:** Oncogenes, DNA damage response

## Abstract

Paraspeckles are mammal-specific membraneless nuclear bodies that participate in various biological processes. NONO, a central paraspeckle component, has been shown to play pivotal roles in DNA double-strand breaks (DSB) repair, whereas its underlying mechanism needs to be further disclosed. Here, using co-immunoprecipitation and mass spectrum, we identified ribosomal protein P0 (RPLP0) as a DSB-induced NONO-binding protein; RPLP0 binds to the RRM1 and RRM2 domains of NONO. Similar to NONO, RPLP0 enhances non-homologous end joining-mediated DSB repair, which was ascribed to a ribosome-independent manner. Interestingly, paraspeckles were induced as early as 15 min after irradiation; it further recruited nuclear RPLP0 to enhance its interaction with NONO. Radiation-induced NONO/RPLP0 complex subsequently anchored at the damaged DNA and increased the autophosphorylation of DNA-PK at Thr2609, thereby enhancing DSB repair. Consistently, in vivo and in vitro experiments showed that depletion of NONO sensitizes tumor cells to radiation. For patients with locally advanced rectal cancer, NONO expression was remarkably increased in tumor tissues and correlated with a poor response to radiochemotherapy. Our findings suggest a pivotal role of radiation-induced paraspeckles in DNA repair and tumor radioresistance, and provide a new insight into the ribosome-independent function of ribosomal proteins.

## Introduction

As one of the major cancer treatment strategies, radiotherapy induces the apoptosis or senescence of tumor cells through triggering a large amount of DNA damage [1–3]. Although radiation could cause different types of DNA damage, including oxidized bases, abasic sites, single-strand breaks, and double-strand DNA breaks (DSBs), DSB is the major radio-toxic damage for live cells [4]. Canonically, DSBs are repaired either by non-homologous end-joining (NHEJ), an error-prone pathway that links the two ends of broken DNA by direct ligation, or by homologous recombination (HR), an error-free mechanism that relies on the presence of a correct template sequence on the sister chromatid [5, 6]. In NHEJ, DSBs are recognized by a heterodimer formed by Ku70 and Ku80. Ku70/80 binds to DNA ends and forms a ring-like structure, which stabilizes the ends of the DSB and recruits factors for NHEJ, including 53BP1 and DNA-dependent protein kinase (DNA-PK) [7–9]. Upon locating to the DSB, the activated DNA-PK phosphorylates several substrates, including DNA ligase IV, XRCC4, and XLF, that are involved in the ligation of DNA ends [10–12].

Paraspeckles are mammal-specific membraneless nuclear bodies that show liquid-like properties and are physically associated with nuclear speckles [13, 14]. Paraspeckles are known to contain over 40 different proteins (most of them are RNA-binding proteins) and one lncRNA, NEAT1 [15]. The function of paraspeckles is not well understood, although they are thought to be involved in transcriptional regulation, mRNA nuclear retention, proteins sequesteration, and microRNA processing [14]. The binding of NONO/SFPQ to NEAT1 is critical for the formation of paraspeckle [16], which can be induced by different cellular stresses [17], including DNA damage [18]. NONO, an essential component of paraspeckle, has also been reported to be involved in the repair of different types of DNA damage. Under ultraviolet microirradiation, NONO was observed to bind to damaged DNA sites within 2–5 min in a PARP1-dependent manner [19, 20], and this process could be abolished by PARP inhibitor [21]. Moreover, NONO deficiency sensitized cells to cisplatin-induced DNA damage, whereas attenuation of XLF expression suppressed the NONO-deficient phenotype [22]. Although NONO-mediated DNA repair has been observed under ultraviolet microirradiation [19, 20] and cisplatin-induced DNA damage [22], whether NONO is involved in X-ray irradiation-induced DSB repair and tumor radioresistance remains undisclosed.

In this study, we found that NONO, an important paraspeckle component, promoted NHEJ-mediated DSB repair and tumor radioresistance. Further investigation identified RPLP0, a ribosomal protein, as an irradiation-induced NONO-binding protein and an enhancer of DSB repair. Interestingly, paraspeckles were largely induced as early as 15 min after irradiation, followed by recruiting RPLP0 to enhance its interaction with NONO. Radiation-induced NONO/RPLP0 complex binds to DSB and promotes DNA-PK autophosphorylation at T2609, leading to tumor radioresistance. These results provide new insights into paraspeckle-mediated tumor radioresistance, which in turn provides a promising molecular target to sensitize rectal cancer cells to radiotherapy.

## Results

### Irradiation increases the interaction between NONO and ribosomal protein P0 in nucleus

**Fig. 1:**
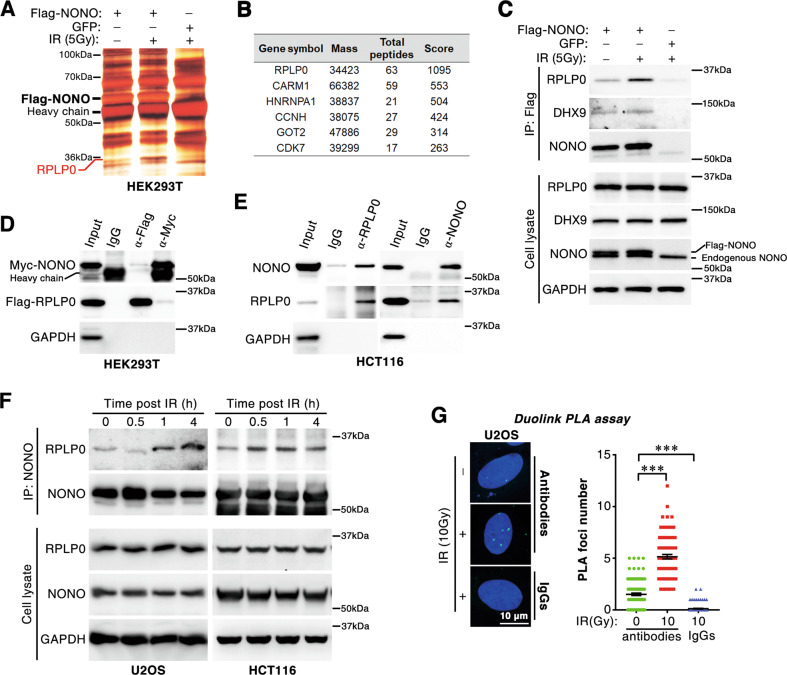
Irradiation increases the association between NONO and ribosomal protein RPLP0. **A** Screening of IR-induced NONO-binding protein. HEK 293T cells were transfected with indicated plasmid and treated with IR (5 Gy). After recovery (30 min), the protein interacting with NONO was assessed by CoIP, separated with SDS-PAGE, and identified with LC/MS assay. GFP was used as a negative control. **B** The differentially expressed band at 36 kDa in Fig. 1A was analyzed by LC/MS. **C** The sample in Fig. 1A was analyzed with western blotting. **D** Flag-RPLP0 and Myc-NONO were interacted with each other in HEK 293T cells. Twenty-four hours after transfection with Flag-RPLP0 and Myc-NONO plasmid, cells were subjected to CoIP assay with anti-Myc or anti-Flag antibodies. **E** endogenous RPLP0 and NONO are associated in HCT116 cells. NONO or RPLP0-binding protein was assessed by CoIP assay and analyzed using western blotting. **F** IR enhances the interaction of NONO and RPLP0. HCT116 or U2OS cells were treated with IR (10 Gy) and recovered for indicated time before CoIP assay. **G** Duolink PLA assay was performed to analyze radiation-induced interaction between NONO and RPLP0. Cells were irradiated with 10 Gy X-ray and cultured for 1 h before PLA assay. *n* = 130 (0 Gy), *n* = 90 (10 Gy, antibodies), *n* = 109 (10 Gy, IgG). ***, *P* < 0.001.

As we previously reported, NONO enhanced non-homologous end joining-mediated DSB repair [23] (Fig. S1A–D). We hypothesized that NONO may bind to DNA repair factors upon DSB formation. To further elucidate the mechanism underlying NONO-mediated DSB repair, a co-immunoprecipitation (CoIP) assay was conducted to identify the change in NONO interactome upon X-ray irradiation. The Flag-NONO-immunoprecipitates were separated by SDS-PAGE, and a radiation-induced band observed at ~36 kDa (Fig. 1A) was cut out and analyzed by LC/MS (Fig. 1B). Interestingly, ribosomal protein P0 (RPLP0) was identified as a novel NONO-binding protein, and its association was found to have remarkably increased after irradiation (Fig. 1C). Furthermore, the interaction between NONO and RPLP0 was verified using CoIP assay. Flag-RPLP0 and Myc-NONO interacted reciprocally in co-expressing HEK 293T cells (Fig. 1D). Similarly, the binding between endogenous RPLP0 and NONO was observed in HCT116 and U2OS cells (Fig. 1E and Fig. S2A), and radiation treatment largely enhanced their interaction (Fig. 1F). In addition, the Duolink Proximal Ligation Assay (PLA) assay was performed to visualize the interaction between NONO and RPLP0 in situ, which showed that radiation induced the interaction between NONO and RPLP0 in the nucleus (Fig. 1G and Fig. S2B).

**Fig. 2:**
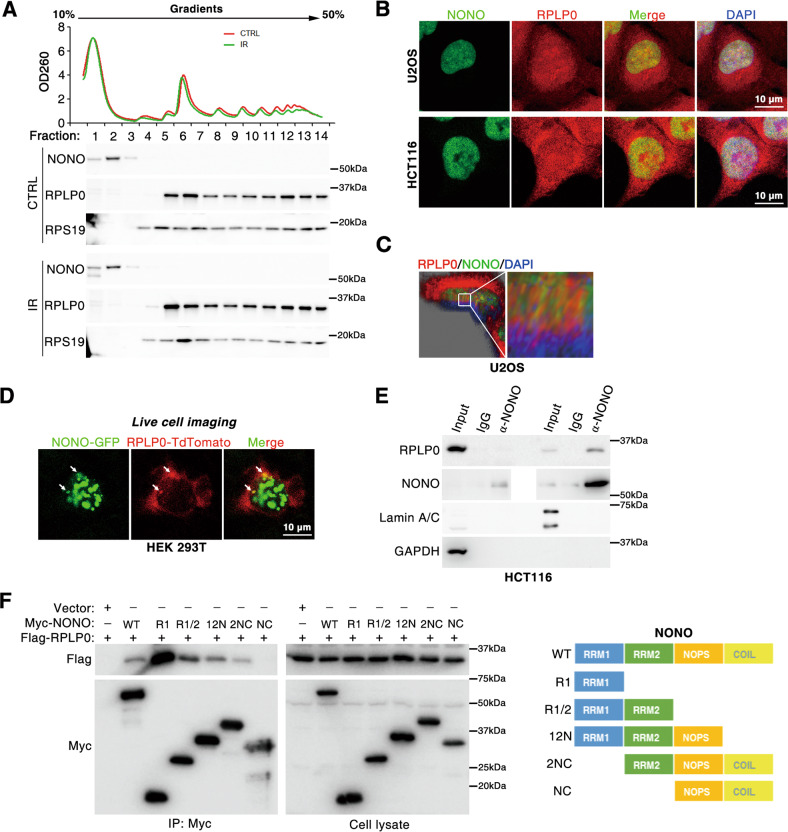
Nuclear RPLP0 interacts with the RRM1/2 domain of NONO. **A** NONO was not detected in ribosome. Ribosomal protein of HCT116 cells with or without IR was fractionated with sucrose density gradient ultracentrifugation. **B** The colocalization between NONO and RPLP0 was analyzed with immunofluorescence. **C** Z-stacking imaging confirmed the association between RPLP0 and NONO. **D** RPLP0-tdTomato and NONO-GFP colocalize in HEK 293T cells. Twenty-four hours after transfection with RPLP0-tdTomato and NONO-GFP plasmids, HEK 293T live cells were imaged using confocal microscopy. **E** RPLP0 and NONO interact in nucleus. Cytoplasmic and nuclear proteins were fractionated and subjected to CoIP assay with anti-NONO antibody. GAPDH and Lamin A/C served as negative controls that did not interact with NONO for cytoplasmic and nuclear extracts, respectively. **F** RRM1 and RRM2 domains of NONO associate with RPLP0. HEK 293T cells were transfected with different mutants of Myc-NONO and Flag-RPLP0 before CoIP assay.

As RPLP0 is a component of the ribosomal large subunit, we further investigated whether NONO bound to ribosomes. Ribosomes were fractionated by sucrose gradient ultracentrifugation and subjected to western blotting. Interestingly, NONO was not detected in the ribosomal fraction of cells with or without IR treatment, indicating that NONO was not associated with ribosomes (Fig. 2A). In contrast, both NONO and RPLP0 were detected in the non-ribosomal fraction (*lanes* 1 and 2, Fig. 2A), suggesting that may be the free RPLP0 protein, rather than that in the ribosome, interacts with NONO. In line with this observation, immunofluorescence (IF) assay (Fig. 2B, C and Fig. S2C) and live cell imaging (Fig. 2D and Fig. S2D) further showed that NONO was colocalized with RPLP0 in the nucleus. Furthermore, the cytoplasmic and nuclear proteins of HCT116 cells were fractionated and subjected to CoIP assay using anti-NONO antibody. As expected, NONO protein was only detected in the nuclear fraction, whereas RPLP0 was present in both the cytoplasmic and nuclear fractions (Fig. 2E). RPLP0 was found to be co-immunoprecipitated with NONO in the nuclear fraction, but not in the cytoplasmic fraction (Fig. 2E). Specifically, the RRM1 and RRM2 domains of NONO were mapped to be responsible for their interactions (Fig. 2F). Together, these results indicate that in the nucleus, RPLP0 binds to the RRM1and RRM2 domains of NONO.

### Radiation-enhanced paraspeckle formation promotes the interaction between NONO and RPLP0

**Fig. 3:**
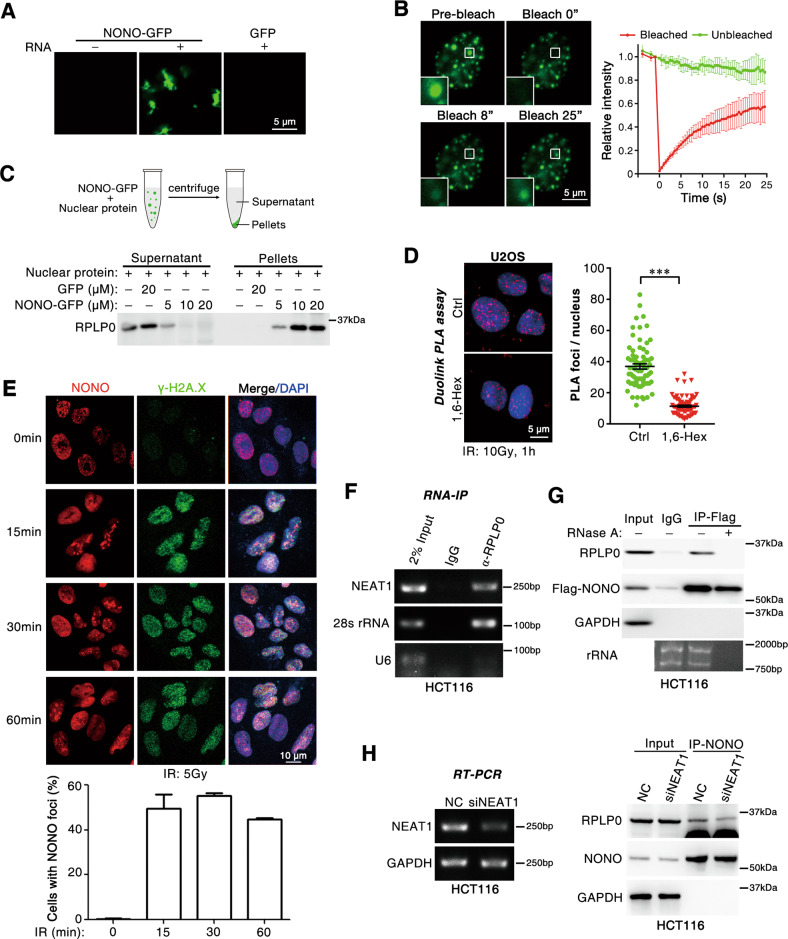
IR-induced paraspeckles recruit RPLP0 and enhances the interaction of NONO and RPLP0. **A** Purified NONO-GFP protein formed condensates in vitro. Ten μM NONO protein and 100 ng/ul total RNA extracted from HEK 293T cells were used. Scale bars, 5 μm. **B** NONO-GFP form condensates with high rate of FRAP in vivo. *n* = 3 biological replicates. Scale bars, 5 μm. **C** NONO condensates recruit RPLP0 protein in vitro. Purified NONO-GFP protein formed condensates in vitro, which were then incubated with nuclear proteins of U2OS cells for 10 min. NONO condensates were separated by centrifugation and subjected to western blotting analysis. **D** 1,6-Hexanediol disrupts the association between NONO and RPLP0. Cells were irradiated (10 Gy) and cultured with complete medium containing 1.5% 1,6-Hexanediol for 30 min before Duolink PLA assay. *n* = 74 (Ctrl), *n* = 78 (1,6-Hex). Scale bars, 5 μm. **E** IR induces paraspeckle formation. U2OS cells were treated with radiation (5 Gy) and allowed to recover for indicated time before IF assay. *n* = 3 biological replicates. Scale bars, 10 μm. **F** RNA-immunoprecipitation assay showed that RPLP0 was associated with NEAT1. 28s rRNA and U6 were used as the positive and negative control, respectively. **G** RNase A disrupts the association between NONO and RPLP0. RNase A (0.1 μg/μl) was added to U2OS cell lysate before CoIP assay. To verify the RNase A-mediated removing of RNA, total RNA was extracted from 10% of flow through lysate and analyzed with gel electrophoresis. **H** siNEAT1 reduced the interaction between NONO and RPLP0. HCT116 cells were transfected with siNEAT1 (pool of siNEAT1-1 and siNEAT1-2) for 48 h before CoIP (*right panel*) or RT-PCR (*left panel*) analysis. ***, *P* < 0.001.

NONO is a well-known component of paraspeckle, a membraneless organelle driven by liquid–liquid phase separation (LLPS). Hence, we asked whether paraspeckles recruited RPLP0 to facilitate the association between NONO and RPLP0 in response to irradiation. When incubated with the total RNA extracted from HEK 293T cells, NONO-GFP protein underwent phase separation, whereas it remained soluble without RNA (Fig. 3A and Fig. S3A). In cells, NONO proteins formed highly concentrated droplets containing a high rate of fluorescence recovery after photobleaching (FRAP) (Fig. 3B). This result was in agreement with the essential LLPS hallmark of internal dynamic reorganization and rapid exchange kinetics between condensates and surrounding dilute phase. After incubation with nuclear proteins, in vitro formed NONO condensates were separated via centrifugation and subjected to western blotting analysis. Interestingly, almost all RPLP0 protein in the cell lysate was recruited into NONO condensates (Fig. 3C). Furthermore, 1,6-Hexanediol, a compound known to disrupt LLPS, significantly inhibited radiation-induced NONO/RPLP0 complex (Fig. 3D), whereas it has no impact on MRE11/RAD50 complex (Fig. S3B), indicating that NONO condensates may recruit RPLP0 in vivo. Consistent with the induction of interaction between NONO and RPLP0 by irradiation, paraspeckles, which were represented as NONO foci, remarkably increased after irradiation (Fig. 3E). Besides, we found that RPLP0 was also associated with NEAT1 (Fig. 3F), a long non-coding RNA interacted with NONO and required for paraspeckle formation. Disruption of RNA with RNase A or silencing NEAT1 significantly decreased the association between NONO and RPLP0 (Fig. 3G, H). These results indicate that irradiation promotes paraspeckle formation to recruit RPLP0, thereby enhancing the interaction between NONO and RPLP0.

### RPLP0 promotes NHEJ-mediated DSB repair

**Fig. 4:**
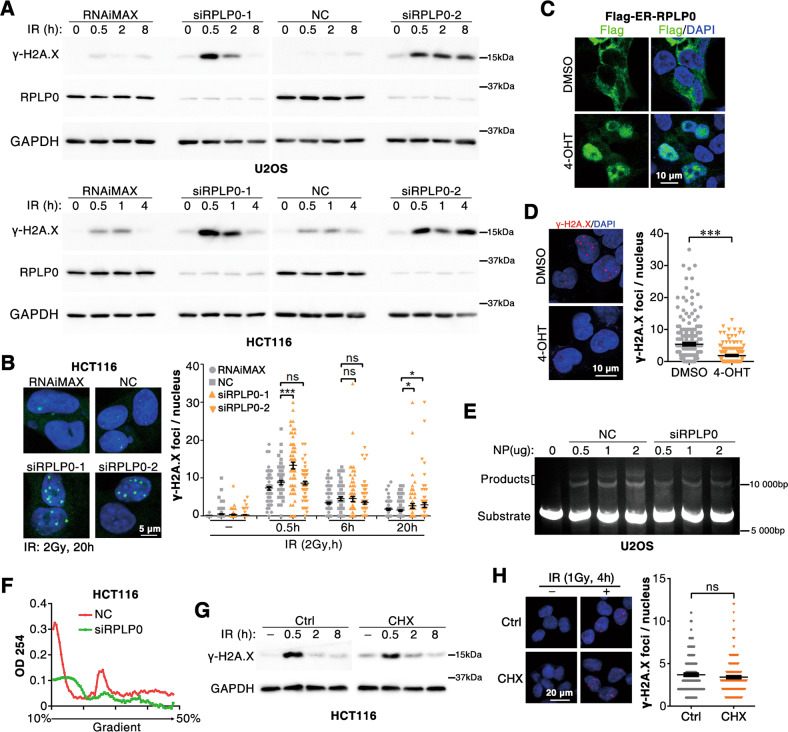
RPLP0 promotes NHEJ-mediated DSB repair. **A** Knockdown of RPLP0 extends the recovery time of IR-induced DNA damage. Forty-eight hours after siRNA transfection, HCT116 and U2OS cells were irradiated (10 Gy), and cultured for indicated time before western blotting analysis. **B** Knockdown of RPLP0 increases γ-H2A.X foci in tumor cells after IR. Thirty-six hours after transfection, HCT116 cells were irradiated (2 Gy) and cultured for indicated time. Cells were fixed and subjected to immunofluorescence analysis. *n* = 120, 85, 59, 96, 73, 58, 62, 75, 96, 62, 68, 93, 71, 81, 64, and 68 (from left to right column). Scale bars, 10 μm. **C** 4-OHT induces the nuclear import of ER-RPLP0. Cells were treated with 300 nM 4-OHT for 4 h before IF assay. Scale bars, 10 μm. **D** Nuclear RPLP0 enhances DNA repair. Thirty minutes before irradiation (2 Gy), 300 nM 4-OHT was added to medium. Cells were cultured for 20 h after irradiation. *n* = 203 (DMSO), *n* = 170 (4-OHT). Scale bars, 10 μm. **E** Depletion of RPLP0 suppresses the ligation of linearized DNA in vitro. pCSCMV-tdTomato plasmid was linearized with BamHI and incubated with nuclear protein of U2OS cells. The ligation product was separated using 0.8% agarose gels. **F** RPLP0 silencing suppresses translation. Forty-eight hours after siRNA transfection, cells were subjected to polysome profile assay. The impact of translation inhibitor cycloheximide (CHX) on DNA repair capacity was examined using western blotting (**G**) or IF assay (**H**). HCT116 cells, treated with 100 μg/ml CHX for 12 h, were irradiated and cultured for indicated time before western blotting (**G**) or IF assay (**H**). *n* = 148 (Ctrl), *n* = 183 (CHX). ***, *P* < 0.001; ns no significance.

We and others have shown that NONO promote NHEJ-mediated DSB repair. We, therefore, hypothesized that RPLP0, an irradiation-induced NONO-binding protein, may also participate in the DSB repair. As expected, silencing RPLP0 via small interfering RNA (siRNA) significantly increased the levels and foci number of radiation-induced γ-H2A.X (Fig. 4A, B and Fig. S4A), suggesting that RPLP0-silencing reduced DNA repair capacity. To overexpress RPLP0 in the nucleus, we constructed ER-RPLP0 fusion protein, which remained in cytoplasm in normal cells but could move into the nucleus upon 4-OHT stimulation [24] (Fig. 4C). Twenty hours after irradiation, 4-OHT treated ER-RPLP0 expressing cells displayed fewer γ-H2A.X foci than the control cells (Fig. 4D). Furthermore, an in vitro NHEJ assay was conducted by incubating the linearized plasmid DNA with the nuclear proteins from control (NC-) or siRPLP0-transfected U2OS cells. As shown in Fig. 4E, compared with NEP from NC-transfected cells, the ligated DNA product of NEP from siRPLP0-transfected cells was remarkably reduced. Because RPLP0 silencing inhibited the assembly of ribosomes (Fig. 4F), we next asked whether siRPLP0-induced translation inhibition had an impact on DNA damage repair. Interestingly, cells treated with cycloheximide (CHX), a translation inhibitor (Fig. S4B), showed similar DNA repair capacity as that of untreated cells (Fig. 4G, H), indicating that RPLP0 functions in NHEJ-mediated DNA repair through a ribosome-independent manner.

### NONO and RPLP0 bind to damaged DNA and increase the phosphorylation of DNA-PK at T2609

**Fig. 5:**
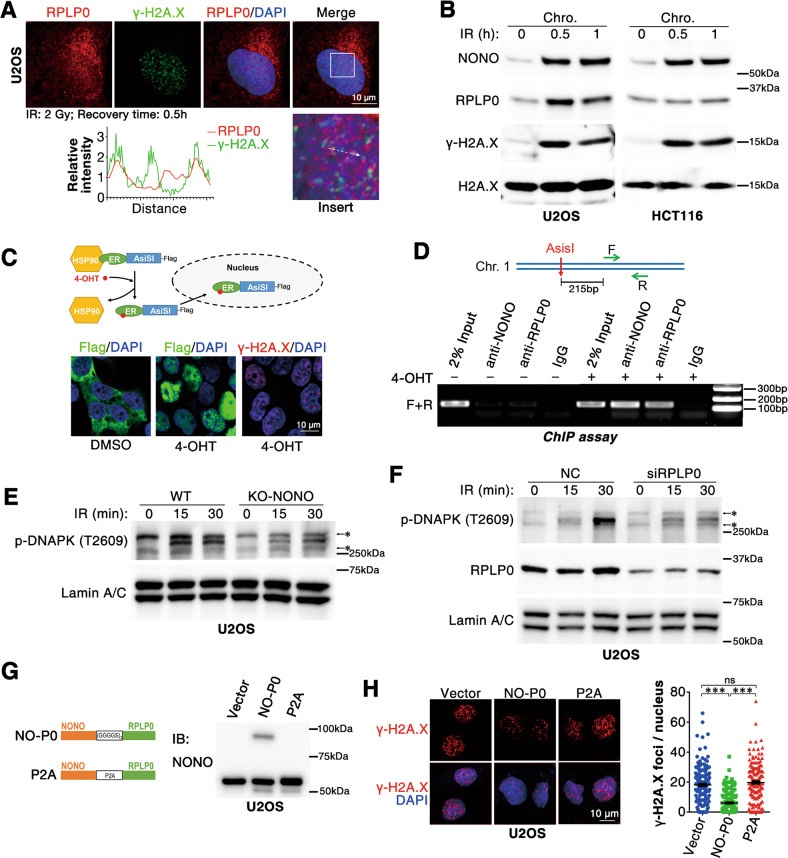
NONO and RPLP0 bind to damaged DNA and increase the phosphorylation of DNA-PK at pT2609. **A** RPLP0 colocalizes with γ-H2A.X after irradiation. U2OS cells were irradiated (2 Gy) and cultured for 30 min before analysis. Scale bars, 10 μm. **B** NONO and RPLP0 load into chromosome upon DNA damage. Twelve hours after seeding, cells were treated with radiation (10 Gy) and cultured for indicated time before chromosome fractionation. **C** 4-OHT induces DNA damage by facilitating the nuclear import of endonuclease ER-AsiSI. Cells were treated with 300 nM 4-OHT for 4 h before IF assay. Scale bars, 10 μm. **D** The binding of NONO and RPLP0 to damaged DNA was examined with ChIP assay. Four hours after 4-OHT or DMSO treatment, ER-AsiSI-expressing U2OS cells were analyzed with ChIP assay. **E**, **F** NONO depletion or RPLP0 silencing inhibits the phosphorylation of DNA-PK at T2609. U2OS cells were irradiated (10 Gy) and cultured for indicated time before nuclei isolation, which were further analyzed by western blotting. *, unspecific band. **G** The construction of NONO-RPLP0 fusion protein. (GGGGS)_3_, triplication of GGGGS; P2A, GSGATNFSLLKQAGDVEENPGP. **H** NONO-RPLP0 fusion protein promotes DNA repair. *n* (Vector) = 178 nuclei, *n* (NO-P0) = 199 nuclei, *n* (P2A) = 179 nuclei. Scale bars, 10 μm. ***; *P* < 0.001; ns no significance.

We further characterized the roles of the NONO/RPLP0 complex in DNA repair. As shown, RPLP0 colocalized with γ-H2A.X foci upon irradiation (Fig. 5A and Fig. S4C, D). Moreover, radiation moderately increased the levels of RPLP0 and NONO in the chromatin fraction (Fig. 5B), indicating that RPLP0 and NONO may bind to damaged DNA. Furthermore, ER-Asi*S*I-expressing cells were treated with 4-OHT to induce DSB before subjecting to chromatin immunoprecipitation (ChIP) assay (Fig. 5C). The result showed that RPLP0 and NONO bound to the Asi*S*I-induced DSB end (Fig. 5D). We and other group [25] have found that NONO promotes the autophosphorylation of DNA-PK at T2609, which is essential for NHEJ-mediated DSB repair. We, therefore, asked whether NONO/RPLP0 regulated pT2609-DNA-PK level. Consistently, RPLP0 silencing significantly inhibited radiation-induced DNA-PK autophosphorylation at T2609 in tumor cells (Fig. 5F), which photocopied RPLP0 silencing (Fig. 5E), indicating that the NONO/RPLP0 complex may promote NHEJ by enhancing DNA-PK autophosphorylation at T2609.

To investigate the function of the NONO-RPLP0 complex in DNA repair and radioresistance, we constructed a NONO-RPLP0 fusion protein linked with a flexible linker [(GGGGS)×3] to mimic the interaction between NONO and RPLP0, and a fusion protein linked with P2A peptide (a peptide with high self-cleavage efficiency) to be used as a negative control [26] (Fig. 5G and Fig. S4E). Interestingly, overexpression of NONO-RPLP0 fusion protein significantly enhanced DNA damage repair, whereas expression of NONO-P2A-RPLP0 had no impact (Fig. 5H), suggesting that NONO/RPLP0 complex enhanced the repair of radiation-induced DNA damage.

### NONO/RPLP0 enhance radioresistance of rectal cancer

**Fig. 6:**
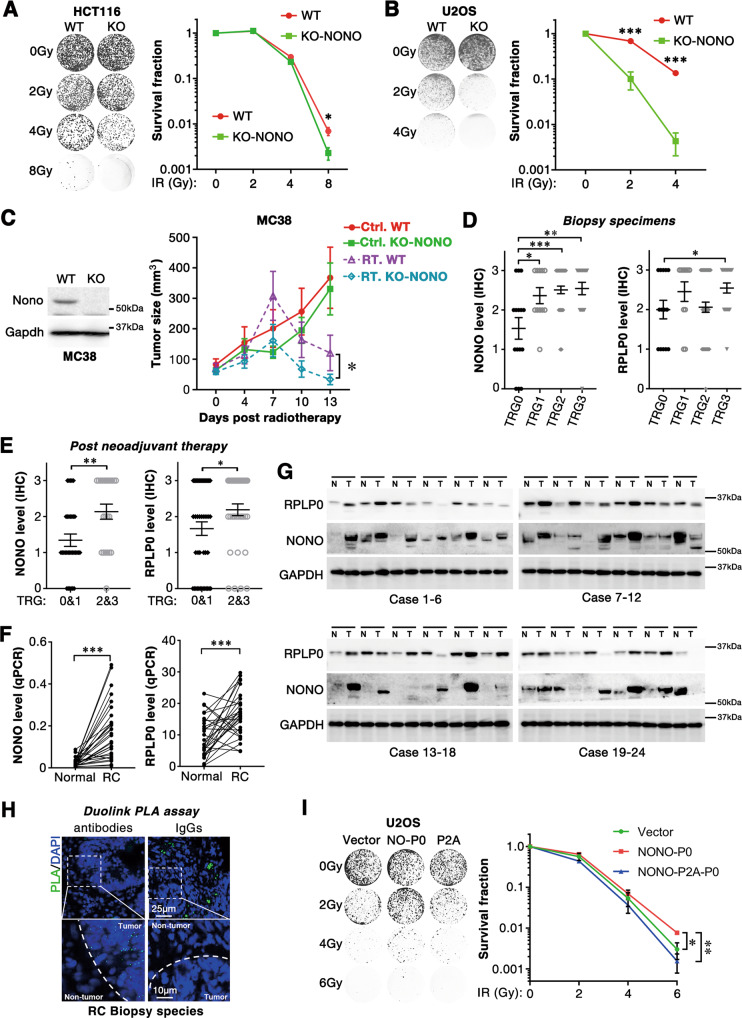
NONO and RPLP0 promote tumor radioresistance. Knocking out of NONO sensitized tumor cells to irradiation. Twenty-four hours after seeding, HCT116 (5000 cells) (**A**) and U2OS (3000 cells) (**B**) were irradiated with indicated dose and cultured for 10 days. Cells were fixed and stained with 0.2% crystal violet. *n* = 3 independent experiments. **C** Knocking out of NONO sensitized xenografts to radiation in vivo. Ten days after MC38 cells (2 × 10^5^) injection, tumors were irradiated with or without 10 Gy (day 0) X-ray and observed for 13 days. RT., Radiotherapy; Ctrl., Control. *n* = 6 xenografts**. D** The NONO and RPLP0 levels in biopsy were analyzed by IHC. *n* = 15 (TRG0), 11 (TRG1), 49 (TRG2), or 22 (TRG3) specimens of rectal cancer. **E** The NONO and RPLP0 levels in operation specimens were analyzed by IHC. *n* = 45 (TRG0&1) or 37 (TRG2&3) specimens of rectal cancer. **F**, **G** The mRNA and protein level of NONO and RPLP0 were examined in rectal cancer tissues. The mRNA and protein level of NONO and RPLP0 in 30 pairs of normal and rectal cancer tissues were examined by qPCR and western blotting analysis. *n* = 30 pairs of normal and rectal cancer tissues. **H** The association of NONO and RPLP0 was examined with Duolink PLA assay in rectal cancer biopsy species. IgGs was used as a negative control. (Top) Scale bars, 25 μm; (Bottom) Scale bars, 10 μm. antibodies: anti-NONO and anti-RPLP0 antibodies; IgGs: immunoglobulin G from mouse and rabbit. **I** NONO-RPLP0 fusion protein promotes radioresistance. *n* = 3 biological replicates. *, *P* < 0.05; **; *P* < 0.01, ***; *P* < 0.001.

Accelerated DNA damage repair is the main cause of tumor radioresistance. We then hypothesized that NONO/RPLP0 may enhance tumor radioresistance. Consistent with the role of NONO in DSB repair, colony formation assay showed that NONO-knockout (NONO-KO) remarkably sensitized HCT116 and U2OS cells to X-ray irradiation (Fig. 6A, B). Furthermore, in vivo xenograft model showed that although NONO depletion had no impact on the growth of MC38 (a mouse colon cancer cell line) xenografts in the control group, NONO-KO MC38 cells-derived xenografts remained smaller than that of wildtype cells in radiotherapy group, indicating that depletion of NONO sensitized tumor cells to radiotherapy (Fig. 6C).

We further evaluated the clinical significance of NONO and RPLP0 in patients with locally advanced rectal cancer (LARC). The protein levels of NONO and RPLP0 were analyzed in biopsy specimens from LARC patients receiving radiochemotherapy. Compared with tumor tissues from patients sensitive to radiochemotherapy (TRG0, tumor regression grades 0), both NONO and RPLP0 proteins were highly expressed in that from resistant patients (TRG3) (Fig. 6D and Fig. S5). Additionally, data from tissues post neoadjuvant therapy showed a similar result (Fig. 6E). Furthermore, compared with normal adjacent tissues, both mRNA and protein levels of NONO were observed to be upregulated in rectal cancer tissues (Fig. 6F, G). Meanwhile, although the mRNA level of RPLP0 was upregulated in the most of rectal cancer tissues, the protein level of RPLP0 only increased in 13/24 tissues (Fig. 6F, G). Interestingly, the Duolink PLA assay in specimens obtained from patients with rectal cancer showed that the binding between NONO and RPLP0 was mainly observed in the nuclei of tumor cells, but not in those of adjacent non-tumor cells (Fig. 6H). Consistently, overexpressing NONO-RPLP0 fusion protein promoted the radioresistance of cancer cells (Fig. 6I). Additionally, the TCGA database showed that RPLP0 and NONO were highly expressed in a variety of cancer types (Fig. S6A), and survival analysis confirmed that high expression of RPLP0 and NONO was associated with an inferior prognosis of cancer patients (Fig. S6B, C).

Together, these data suggest that IR-induced NONO/RPLP0 complex promotes DSB repair and tumor radioresistance by binding to damaged DNA and enhancing the phosphorylation of DNA-PK at T2609.

## Discussion

Radioresistance remains a major cause of cancer-related mortality. However, the underlying mechanisms remain unclear. In this study, we found that NONO, an essential component of paraspeckles, enhanced tumor radioresistance. In particular, we identified ribosomal protein RPLP0 as a novel DNA damage-induced NONO-interacting protein. Upon irradiation, NONO and RPLP0 are recruited to the damaged DNA end to increase DNA-PK phosphorylation at T2609, thereby enhancing NHEJ-mediated DSB repair and inducing tumor cell radioresistance.

NONO belongs to the Drosophila behavior human splicing family, which includes the paralogs SFPQ and PSPC1 [27]. NONO has been reported to be a multifunctional protein, as it regulates gene expression at the transcriptional and post-transcriptional levels such as transcription activation, RNA splicing, and stabilization [28, 29]. In addition, NONO and long noncoding RNA NEAT1 form paraspeckles [16]. Consistent with our findings, NONO has been reported to be involved in different DNA repair pathways. In UV-induced DNA damage response, RNF8-mediated degradation of NONO was found to be required for S phase progression by terminating ATR-CHK1 checkpoint signaling [30, 31]. During DSB repair, depletion of NONO reduces DNA end ligation in NHEJ-core factor-based in vitro ligation assay [19, 22, 32]. The NONO/SFPQ heterodimer has been reported to interact with many DNA repair factors, including RAD51, Ku, TopBP1, and Matrin3 [20, 29]. Our findings also showed that NONO could repair X-ray radiation-induced DSBs via NHEJ. Although many NHEJ factors have been identified as NONO partners, most of these associations were not affected by radiation [20], hinting at a largely unknown part of the underlying regulatory mechanisms. Here, we characterized RPLP0 as a NONO-interacting protein, and their association was significantly enhanced by X-ray irradiation, indicating that RPLP0 may function as a transductor that activates NONO-associated DNA repair components upon X-ray-induced DNA damage in cancer cells.

A previous study has linked paraspeckles with replication stress response and chemosensitivity. Adriaens et al. reported that paraspeckle enhancing HU-induced ATR activation, thereby preventing DNA damage from replication stress or chemotherapy [18]. In this study, authors found that p53 could induce the formation of paraspeckles by upregulating NEAT1. However, our data showed that radiation-induced paraspeckle formation could be detected as early as 5 min after irradiation, which is much earlier than the activation of p53 after irradiation. Hence, other mechanisms, but not p53-induced NEAT1 upregulation, may underlie the radiation-induced paraspeckle formation at early stage. Most importantly, in this study, we disclosed a pivotal function of paraspeckle in DSB repair: paraspeckle recruited RPLP0 to enhance its interaction with the paraspeckle component NONO, thereby promoting DNA-PK phosphorylation at T2609 and enhancing DSB repair.

Although ribosomal proteins are well known for their essential roles in ribosome assembly and protein translation, their ribosome-independent functions have also been characterized and reported in the context of various biological processes, including DNA damage response [33–35]. For example, upon stimulation with reactive oxygen species, ribosomal protein S3 binds to HSP90, HSP70, and TOM70, thereby translocating into mitochondria and repairing reactive oxygen species-induced mitochondrial DNA damage [36–38]. In the present study, we reported that ribosomal protein RPLP0 enhanced DSB repair through the NHEJ pathway. To the best of our knowledge, this is the first report on the participation of ribosomal proteins in DSB repair. Additionally, the oncogenic function of RPLP0 has been previously reported [39]. RPLP0 interacts with GCIP to activate cyclin D1, thereby promoting tumor cell proliferation [40]. Conversely, depletion of RPLP0 leads to apoptosis and cell cycle arrest in cancer cells [41]. In this study, we observed the upregulation of RPLP0 in locally advanced rectal cancer tissues and its higher expression was correlated with radioresistance of rectal cancer patients. Most importantly, we characterized RPLP0 as an important regulator of NHEJ-mediated DSB repair and tumor radioresistance. These results provide new insights into ribosome-independent function of ribosomal protein.

After radiation-induced DSB formation, DNA-PK is rapidly autophosphorylated at T2609, which is essential for its DNA repair activity [42]. However, little is known about the control of DNA-PK phosphorylation. Our previous study has shown that EGFR could translocate into the nucleus after irradiation, bind to DNA-PK, and induce its phosphorylation [23]. Moreover, IGFBP2 was found to translocate into the nucleus and enhanced the association between EGFR and DNA-PK [43]. In this study, we found that ribosomal protein RPLP0 was a new regulator of radiation-induced DNA-PK phosphorylation. Meanwhile, we noticed the limitation of our study that the mechanism underlying how RPLP0/NONO complex enhanced DNA-PK phosphorylation remained undisclosed.

In summary, our data have shown that NONO, a central component of paraspeckles, enhances tumor radioresistance by promoting the repair of radiation-induced DSBs. RPLP0, a ribosomal protein that functions in a ribosome-independent manner, is recruited by irradiation-induced paraspeckles and interacts with NONO in the nucleus, localizes to damaged DNA, and enhances the activation of DNA-PK and NHEJ-dependent DNA repair. These findings introduce new roles of paraspeckles and ribosomal proteins in DNA damage repair, which may provide novel molecular targets for cancer treatment.

## Materials and methods

### Cell lines and tissue specimens

Human colorectal carcinoma cell line HCT116 and human embryonic kidney cell line HEK 293T were purchased from American Type Culture Collection (ATCC). Human osteosarcoma cell line U2OS was purchased from Guangzhou Cellcook Biotech Co., Ltd. Mouse colon cancer cell line MC38 was kindly provided by Prof. Huanliang Liu at Sun Yat-sen University. All cell lines were mycoplasma-free and were authenticated using STR profiling by the provider ATCC or Cellcook. HCT116 and MC38 cells were cultured in RPMI 1640 medium (Gibco, Thermo Fisher Scientific, Waltham, Massachusetts, USA) supplemented with 10% fetal bovine serum (FBS) (Gibco). HEK 293T and U2OS were cultured in Dulbecco’s modified Eagle’s medium (DMEM, Gibco) supplemented with 10% FBS (FBS, Gibco). NONO-knockout HCT116 (HCT116-KO), U2OS (U2OS-KO), and MC38 (MC38-KO) cells were generated using CRISPR/cas9 editing tools.

Human locally advanced rectal cancer and adjacent non-tumor rectal tissues were obtained from the Tissue Bank of the Sixth Affiliated Hospital, Sun Yat-sen University. All patients underwent radical surgery, and both tumor and adjacent normal tissues were histologically confirmed. Informed consent was obtained from each patient, and the protocol was approved by the Institutional Research Ethics Committee of the Sixth Affiliated Hospital, Sun Yat-sen University.

### RNA oligoribonucleotides and vectors

All RNA oligonucleotides were purchased from GenePharma (Shanghai, China). The siRNAs targeting the human RPLP0 and NEAT1 transcripts were designated as siRPLP0 and siNEAT1, respectively. The negative control (NC) RNA duplex for siRNAs was non-homologous to any other human genome sequence. The siRNA sequences were provided in Supplementary Table S1.

The expression vectors pCDH-Flag-NONO and pCDH-Flag-RPLP0 were produced by inserting C-terminal Flag-tagged NONO and RPLP0 sequences, respectively into pCDH-CMV-MCS-EF1-copGFP vectors (pCDH, System Biosciences, Palo Alto, CA, USA). To express NONO-linker-RPLP0 fusion proteins, the coding sequences of NONO, linker, and RPLP0 were sequentially inserted into pCDH vector to construct expression vector. The amino acid sequence of linkers: (GGGGS)_3_, GGGGSGGGGSGGGGS; P2A, GSGATNFSLLKQAGDVEENPGP.

To generate NONO-knockout cell lines, lenti-CRISPR-sg-NONO vector was produced by inserting 5′-GAGTAATAAAACTTTTAACT-3′ (Human) or 5′-TCTTCCCCCTGATATCACTG-3′ (Mouse) sequence into the *BsmB*I site of lenti-CRISPR-v2 (Addgene, Watertown, MA, USA).

pCDH-ER-AsiSI vector was used to introduce site-specific DSB in cells. Ligand binding domain of ER (containing C400V/M543A/L544A site mutations) and AsiSI coding sequence was obtained from Genebank and synthesized by GENEWIZ (Soochow, China). A nuclear localization signal (NLS) and a Flag tag were added to the C-terminal of ER-AsiSI using PCR, and inserted into pCDH to generate pCDH-ER-AsiSI vector.

pCDH-ER-RPLP0 vector expressing ER-RPLP0-NLS-Flag was generated as pCDH-ER-AsiSI vector.

### Analysis of gene expression

Real-time quantitative polymerase chain reaction (qPCR) assays were performed to evaluate RNA levels. Total RNA was extracted using TRIzol reagent (Invitrogen, Thermo Fisher Scientific, Waltham, Massachusetts, USA) and reverse-transcribed using PrimeScript™ RT reagent Kit with gDNA Eraser (RR047A, TaKaRa, Kyoto, Japan). qPCR was performed on ABI7500 (Applied Biosystems, Waltham, Massachusetts, USA) or LightCycler480 (Roche Diagnostics, Germany) using 2×SYBR Green qPCR Master Mix (QPK-201, TOYOBO, Kita-ku, Osaka, Japan). All reactions were performed in duplicates. The cycle threshold (Ct) values between duplicate wells differed by less than 0.5. The relative expression levels of the target genes were normalized to those of the internal control genes, which yielded the 2^−ΔCt^ value. U6 and GAPDH were used as reference genes for relative expression levels in tissues and cell lines, respectively.

Western blotting was performed to determine protein levels. The antibodies used were as follows: anti-GAPDH (60004-1-Ig, Proteintech, Rosemont, IL, USA), anti-NONO (611279, BD Bioscience, Franklin Lakes, NJ, USA), anti-RPLP0 (A5557, Abclonal, Wuhan, China), anti-Flag (ab49763, Abcam, Cambridge, MA, USA), anti-Myc (ab1326, Abcam), anti-RPS19 (A2019, Abclonal), anti-γ-H2A.X (9718, Cell Signaling Technology, CST, Beverly, MA, USA), anti-H2A.X (A11412, Abclonal), anti-Lamin A + C (ab108595, Abcam), anti-α-tubulin (66031-1-Ig, Proteintech).

Immunohistochemical (IHC) analysis was carried out to analyze the protein levels in the tissue. Briefly, formalin-fixed paraffin-embedded tissue was cut into 5 μm sections, placed on polylysine-coated slides, de-paraffinized in xylene, and rehydrated through graded ethanol. After quenching endogenous peroxidase activity, sections were processed for antigen retrieval by microwave heating in 10 mM citrate buffer (pH 6.0), followed by overnight incubation at 4 °C with RPLP0 (A5557, Abclonal) or NONO (611279, BD Bioscience) antibodies. Immunostaining was performed using Biotin-Streptavidin HRP Detection Systems (SP-9000, ZSGB-BIO, Beijing, China), which resulted in a brown-colored precipitate at the antigen site. The sections were subsequently counterstained with hematoxylin (Zymed Laboratories, South San Francisco, CA) and mounted in a non-aqueous mounting medium. The signal was scored as described previously [44].

### Cell transfection

RNA oligonucleotides and plasmid were respectively transfected using Lipofectamine RNAiMAX (Invitrogen) and Lipofectamine 2000 (Invitrogen). A final concentration of 20 nM siRNA was used.

### Colony formation assay

The radiosensitivity of tumor cells was examined using the colony formation assay. Wild-type or NONO-knockout HCT116 (5000 cells/well) or U2OS (3000 cells/well) cells were placed in a six-well plate, treated with the indicated dose of radiation, and maintained in a complete medium for 10 days. After fixing in methanol, the colonies were stained with 0.1% crystal violet solution in 20% methanol for 15 min. Images were captured using a scanner (Canon) and analyzed using Image J.

### Mouse xenograft models

All animal experiments were approved by the Institutional Animal Care and Use Committee of the Sixth Affiliated Hospital of Sun Yat-sen University (Accreditation No. IACUC-2020072801). MC38 or MC38-KO-Nono cells (2 × 10^5^ in 1 × PBS) were injected subcutaneously into the right or left posterior flank of 5-week-old male C57BL/6 mice (GemPharmatech, Nanjing, China). Two weeks later, The C57BL/6 mice were randomly allocated into irradiation or control group and the investigators were blinded to experiments and outcome assessment. The xenografts in irradiation group were irradiated with a single 5 Gy dose of X-rays. The experiments ended 13 days after irradiation. Tumor volume (V) was monitored by measuring the length (*L*) and width (*W*) with calipers and calculated using the following formula: (*L* × *W*^2^) × 0.5.

### Immunofluorescence (IF)

After indicating treatment, cells were fixed in 4% paraformaldehyde for 15 min, permeabilized with 0.3% Triton X-100 in 1 × PBS for 15 min, blocked with 5% goat serum for 1 h, and incubated with the primary antibody diluted in 1 × PBS containing 5% goat serum for 2 h at room temperature. After three washes with 1 × PBS, cells were incubated with secondary antibody for 1 h, stained with DAPI for 5 min, and mounted in ProLong™ Diamond Antifade Mountant (P36965, Thermo Fisher). Antibodies used for immunofluorescence are as follows: NONO (A3800, Abclonal), RPLP0 (A5557, Abclonal), and γ-H2A.X (9718, CST; 80312S, CST; 05–636, Merck Millipore, Darmstadt, Germany).

### Co-immunoprecipitation (CoIP)

Cells were lysed using IP-lysis buffer [25 mM Tris-HCl at pH 7.4, 150 mM NaCl, 1 mM EDTA, 1% NP-40, 5% glycerol, and protease inhibitor cocktail (04693132001, Roche, Basel, Switzerland)] at 4 °C for 30 min, and the supernatant was collected after centrifugation (13,000 × *g*, 4 °C, 10 min). For immunoprecipitation of Flag-tagged protein, cell lysates were mixed with Anti-FLAG® M2 Magnetic Beads (M8823, Sigma-Aldrich, St. Louis, Missouri, USA) and rotated at 4 °C for 4 h. For immunoprecipitation with other antibodies, cell lysates were mixed with 4 μg antibody or IgG by gently rotation at 4 °C for 4 h, followed by the addition of 40 μl Dynabeads G (10004D, Invitrogen) and further incubation at 4 °C for 2 h with gentle rotation. After four to five washes with IP-lysis buffer, immunoprecipitated proteins were eluted from beads with 50 μl 100 mM glycine (pH 3.5), boiled in 1× SDS loading buffer (50 mM Tris-HCl at pH 6.8, 2% SDS, 0.01% bromophenol blue, 10% glycerol, and 1% 2-mercaptoethanol), resolved on a sodium dodecyl sulfate-polyacrylamide gel (SDS-PAGE) and subjected into immunoblotting or silver staining. After silver staining, the targeted bands were cut out and analyzed with LC/MS (Beijing Protein Innovation, Beijing, China).

### RNA-immunoprecipitation (RNA-IP)

HCT116 cells were lysed with IP-lysis buffer supplemented with 1000 U/ml RNasin ribonuclease inhibitor (Promega, Madison, WI, USA), incubated with 4 μg anti-RPLP0 antibody (11290-2-AP, Proteintech) or IgG, and mixed with Dynabeads G (10004D, Invitrogen), as described in Co-IP assay. After washed with IP-lysis buffer for 5 times, RNA was extracted from beads with Trizol reagent and subjected into Reverse Transcription-PCR analysis.

### Polysome profile

Polysome analysis was performed as described previously [45]. After ultra-centrifugation, the mixture was fractionated and absorbance at 260 nm was recorded using a BioComp Piston Gradient Fractionator equipped with a Bio-Rad Econo UV Monitor. The corresponding fractions were further subjected to western blotting.

### Duolink proximal ligation assay

The sections embedded in paraffin were de-paraffinized in xylene, rehydrated through graded ethanol, and processed for antigen retrieval by microwave heating in 10 mM citrate buffer (pH 6.0). Cells seeded on slides were fixed in 4% paraformaldehyde for 15 min and permeabilized with 0.3% Triton X-100 in 1 × PBS for 15 min. After three washes with 1 × PBS, slides were incubated with blocking buffer (5% goat serum and 0.3% Triton X-100 in 1 × PBS) at room temperature for 1 h, followed by overnight incubation at 4 °C with primary antibodies. After three washes with 1 × PBS, slides were incubated with Duolink® In Situ PLA® Probe Anti-Rabbit MINUS (DUO92005, Sigma-Aldrich) and Duolink® In Situ PLA® Probe Anti-Mouse PLUS (DUO92001, Sigma-Aldrich) at 37 °C for 1 h, followed by ligation and amplification using Duolink® In Situ Detection Reagents. After staining with DAPI, slides were mounted in ProLong™ Diamond Antifade Mountant. Images were acquired using an LSM 880 microscope (Zeiss, Jena, Germany). Primary antibodies used in this assay: anti-RPLP0 (A5557, Abclonal) and anti-NONO (611279, BD Bioscience), anti-MRE11 (ab214, Abcam), and anti-RAD50 (3427S, CST).

### Fractionation of cytoplasmic, nuclear, and chromatin-associated proteins

After two washes with 1 × PBS, the cells were scraped and suspended in 250 μl Cyto-lysis buffer [10 mM HEPES-NaOH, pH 7.9, 10 mM KCl, 1.5 mM MgCl_2_, and 0.5 mM beta-mercaptoethanol, supplemented with protease inhibitor and phosphatase inhibitor cocktail (04906837001, Roche)], vortexed, and incubated on ice for 15 min. After 5 μl 10% NP-40 was added, mixture was incubated on ice for 2 min, and centrifuged at 16,000 × *g*, 4 °C for 10 min. The supernatant was kept as cytoplasmic fraction. The pellet was washed twice with ice-cold 1 × PBS, and 60–80 μl Nucl-lysis buffer (10 mM Tris-HCl, pH 7.6, 420 mM NaCl, 0.5% Nonidet P-40, 1 mM DTT, 1 mM PMSF and 2 mM MgCl_2_, supplemented with protease inhibitor and phosphatase inhibitor cocktail) was then added to the pellet. The pellet was dispersed with tips, placed on ice for 20 min, and centrifuged at 16,000 × *g*, 4 °C for 10–15 min. The supernatant was kept as nucleoplasm fraction. After washing twice with 1 × PBS, pellets were suspended in 80 μl 0.25 M HCl, kept at 4 °C overnight, and centrifuged at 16,000 × *g*, 4 °C for 10 min. This extract contained histone and chromatin-associated proteins. The efficiency of fractionation was confirmed with western blotting.

### In vitro NHEJ assay

The pCSCMV-tdTomato plasmid was linearized with BamHI (R3136, New England Biolabs, Beverly, MA, USA) and used as a substrate. The nuclear proteins of HCT116 or U2OS cells were fractionated as described above. The linearized plasmid was incubated with different amounts of nuclear protein in 20 μl NHEJ buffer (20 mM HEPES-KOH at pH 7.5, 80 mM KCl, 10 mM MgCl_2_, 1 mM ATP, 1 mM DTT, and 1 mM dNTP mix) at 30 °C for 30 min. Reactions were terminated with the addition of 2 μl 0.5 M EDTA, 2 μl 0.5% sodium dodecyl sulfate, and 1 μl 10 mg/mL Proteinase K (EO0491, Thermo Fisher Scientific), followed by incubation at 37 °C for 30 min. The products were separated by gel electrophoresis.

### Protein purification

cDNA encoding NONO was cloned into a modified version of the pGEX-6P-1 vector, which include a C-terminal EGFP. The cDNA sequence of NONO generated by PCR was inserted in-frame before EGFP.

For protein expression, the constructed plasmid pGEX-NONO-GFP or the base vector pGEX-GFP were transformed into *E. coli* strain BL21(DE3) cells. Bacteria were cultured in 300 mL LB medium containing ampicillin and grown at 37 °C in a constant temperature shaker for approximately 7 h. When OD_600_ of the mixture reached 0.6–0.8, IPTG was added to the culture to a final concentration of 0.1 mM. After 12 h, the induced bacteria were collected by centrifugation at 4000 × *g*, 4 °C for 10 min. The pellet was resuspended in 1 × PBS supplemented with 1 mM PMSF, followed by sonication. Protein purification was performed according to the manufacturer’s protocol for the GST-tag protein purification kit (P2262, Beyotime, Shanghai, China).

### In vitro droplets formation

Purified NONO-GFP or GFP proteins were diluted to 10 μM in a buffer containing 150 mM NaCl, 20 mM Tris-HCl (pH = 7.4), with or without 100 ng/ul total RNA extracted from HEK 293T cells. The recombinant protein mixture was incubated at 25 °C for 10 min and then applied to a glass slide. Images were acquired using Zeiss LSM 880 confocal microscope (Zeiss).

### Droplets pelleting

The nuclear proteins from U2OS cells were fractionated as described above. Purified NONO-GFP or GFP proteins were incubated with nuclear proteins for 10 min at room temperature in a buffer containing 150 mM NaCl and 20 mM Tris-HCl (pH = 7.4). The protein mix was then centrifuged at 10,000 × *g*, 4 °C for 10 min, and the supernatant and pellet were stored separately for further analysis. After boiled at 100 °C for 15 min in 1×SDS loading buffer (2% SDS, 10 mM EDTA, 10% glycerol, 0.1% bromophenol blue, 50 mM Tris-HCl, pH6.8), proteins were subjected to western blotting analysis.

### Fluorescence recovery after photobleaching (FRAP)

FRAP was performed using Zeiss LSM 880 confocal microscope (Zeiss). A round bleach spot was chosen inside the droplet, and then the spot was bleached using 100% laser power using a 488 nm laser. Images were acquired using a 63× oil immersion objective every 2 s. Images were further processed and fluorescence intensity were measured using Zeiss ZEN software. The relative intensity was considered to be the ratio of the evaluated intensity to the intensity of pre-bleached spot.

### Statistical analysis

The differences in NONO and RPLP0 expression levels between the paired locally advanced rectal cancer tissues and adjacent normal rectal tissues were compared using paired *t*-test. The differences in NONO and RPLP0 expression levels between patients of different TRG, which defined by the American College of Pathologist Guidelines [46], were compared using unpaired *t*-test.

The data are expressed as the mean ± standard error of the mean (SEM) obtained from at least three independent experiments. The differences between groups were analyzed using an unpaired *t*-test. *P* < 0.05 was considered as statistically significant. All statistical tests were two-sided and were performed using GraphPad Prism (GraphPad Software Inc., San Diego, CA, USA). For colony formation, IF and PLA assay, experiments were blinded to the person performing image analysis.

## Supplementary information

Supplemental figures and table

Original western blots and DNA gels

Reproducibility checklist

## Data Availability

The data used and/or analyzed during the current study are available from the corresponding author on reasonable request. The uncropped western blotting data has been provided in the Supplementary Information.

## References

[1] Goldstein M, Kastan MB (2015). The DNA damage response: Implications for tumor responses to radiation and chemotherapy. Annu Rev Med.

[2] Wang H, Mu X, He H, Zhang XD (2018). Cancer radiosensitizers. Trends Pharmacol Sci.

[3] Wang Y, Boerma M, Zhou D (2016). Ionizing radiation-induced endothelial cell senescence and cardiovascular diseases. Radiat Res.

[4] Lomax ME, Folkes LK, O’Neill P (2013). Biological consequences of radiation-induced DNA damage: Relevance to radiotherapy. Clin Oncol (R Coll Radio).

[5] O’Connor MJ (2015). Targeting the DNA damage response in cancer. Mol Cell.

[6] Caridi CP, Plessner M, Grosse R, Chiolo I (2019). Nuclear actin filaments in DNA repair dynamics. Nat Cell Biol.

[7] Lanz MC, Dibitetto D, Smolka MB (2019). DNA damage kinase signaling: Checkpoint and repair at 30 years. Embo J.

[8] Deshpande RA, Myler LR, Soniat MM, Makharashvili N, Lee L, Lees-Miller SP (2020). DNA-dependent protein kinase promotes DNA end processing by MRN and CtIP. Sci Adv.

[9] Tang M, Feng X, Pei G, Srivastava M, Wang C, Chen Z (2020). FOXK1 participates in DNA damage response by controlling 53BP1 function. Cell Rep.

[10] Ahnesorg P, Smith P, Jackson SP (2006). XLF interacts with the XRCC4-DNA ligase IV complex to promote DNA nonhomologous end-joining. Cell..

[11] Ceccaldi R, Rondinelli B, D’Andrea AD (2016). Repair pathway choices and consequences at the double-strand break. Trends Cell Biol.

[12] Chang HHY, Pannunzio NR, Adachi N, Lieber MR (2017). Non-homologous DNA end joining and alternative pathways to double-strand break repair. Nat Rev Mol Cell Biol.

[13] Nakagawa S, Hirose T (2012). Paraspeckle nuclear bodies–useful uselessness?. Cell Mol Life Sci.

[14] Fox AH, Nakagawa S, Hirose T, Bond CS (2018). Paraspeckles: Where long noncoding RNA meets phase separation. Trends Biochem Sci.

[15] Taiana E, Ronchetti D, Todoerti K, Nobili L, Tassone P, Amodio N, et al. LncRNA NEAT1 in paraspeckles: A structural scaffold for cellular DNA damage response systems? Noncoding RNA. 2020;6:26. 10.3390/ncrna6030026 PMC7549348 32630183

[16] Yamazaki T, Souquere S, Chujo T, Kobelke S, Chong YS, Fox AH (2018). Functional domains of NEAT1 architectural lncRNA induce paraspeckle assembly through phase separation. Mol Cell.

[17] Wang S, Zhang Q, Wang Q, Shen Q, Chen X, Li Z (2018). NEAT1 paraspeckle promotes human hepatocellular carcinoma progression by strengthening IL-6/STAT3 signaling. Oncoimmunology..

[18] Adriaens C, Standaert L, Barra J, Latil M, Verfaillie A, Kalev P (2016). p53 induces formation of NEAT1 lncRNA-containing paraspeckles that modulate replication stress response and chemosensitivity. Nat Med.

[19] Krietsch J, Caron MC, Gagne JP, Ethier C, Vignard J, Vincent M (2012). PARP activation regulates the RNA-binding protein NONO in the DNA damage response to DNA double-strand breaks. Nucleic Acids Res.

[20] Salton M, Lerenthal Y, Wang SY, Chen DJ, Shiloh Y (2010). Involvement of Matrin 3 and SFPQ/NONO in the DNA damage response. Cell Cycle.

[21] de Silva HC, Lin MZ, Phillips L, Martin JL, Baxter RC (2019). IGFBP-3 interacts with NONO and SFPQ in PARP-dependent DNA damage repair in triple-negative breast cancer. Cell Mol Life Sci: CMLS.

[22] Jaafar L, Li Z, Li S, Dynan WS (2017). SFPQ*NONO and XLF function separately and together to promote DNA double-strand break repair via canonical nonhomologous end joining. Nucleic Acids Res.

[23] Fan XJ, Wang YL, Zhao WW, Bai SM, Ma Y, Yin XK (2021). NONO phase separation enhances DNA damage repair by accelerating nuclear EGFR-induced DNA-PK activation. Am J Cancer Res.

[24] Iacovoni JS, Caron P, Lassadi I, Nicolas E, Massip L, Trouche D (2010). High-resolution profiling of gammaH2AX around DNA double strand breaks in the mammalian genome. EMBO J.

[25] Udayakumar D, Dynan WS (2015). Characterization of DNA binding and pairing activities associated with the native SFPQNONO DNA repair protein complex. Biochem Biophys Res Commun.

[26] Kim JH, Lee SR, Li LH, Park HJ, Park JH, Lee KY (2011). High cleavage efficiency of a 2A peptide derived from porcine teschovirus-1 in human cell lines, zebrafish and mice. PLoS One.

[27] Lahaye X, Gentili M, Silvin A, Conrad C, Picard L, Jouve M (2018). NONO detects the nuclear HIV capsid to promote cGAS-mediated innate immune activation. Cell..

[28] Benegiamo G, Mure LS, Erikson G, Le HD, Moriggi E, Brown SA (2018). The RNA-binding protein NONO coordinates hepatic adaptation to feeding. Cell Metab.

[29] Knott GJ, Bond CS, Fox AH (2016). The DBHS proteins SFPQ, NONO, and PSPC1: A multipurpose molecular scaffold. Nucleic Acids Res.

[30] Deshar R, Yoo W, Cho EB, Kim S, Yoon JB (2019). RNF8 mediates NONO degradation following UV-induced DNA damage to properly terminate ATR-CHK1 checkpoint signaling. Nucleic Acids Res.

[31] Alfano L, Costa C, Caporaso A, Altieri A, Indovina P, Macaluso M (2016). NONO regulates the intra-S-phase checkpoint in response to UV radiation. Oncogene..

[32] Bladen CL, Udayakumar D, Takeda Y, Dynan WS (2005). Identification of the polypyrimidine tract binding protein-associated splicing factor.p54(nrb) complex as a candidate DNA double-strand break rejoining factor. J Biol Chem.

[33] Zhou X, Liao WJ, Liao JM, Liao P, Lu H (2015). Ribosomal proteins: Functions beyond the ribosome. J Mol Cell Biol.

[34] Pelletier J, Thomas G, Volarevic S (2018). Ribosome biogenesis in cancer: New players and therapeutic avenues. Nat Rev Cancer.

[35] Goudarzi KM, Lindstrom MS (2016). Role of ribosomal protein mutations in tumor development (Review). Int J Oncol.

[36] Kim Y, Kim HD, Kim J (2013). Cytoplasmic ribosomal protein S3 (rpS3) plays a pivotal role in mitochondrial DNA damage surveillance. Biochim Biophys Acta.

[37] Kim J, Chubatsu LS, Admon A, Stahl J, Fellous R, Linn S (1995). Implication of mammalian ribosomal protein S3 in the processing of DNA damage. J Biol Chem.

[38] Grosheva AS, Zharkov DO, Stahl J, Gopanenko AV, Tupikin AE, Kabilov MR (2017). Recognition but no repair of abasic site in single-stranded DNA by human ribosomal uS3 protein residing within intact 40S subunit. Nucleic Acids Res.

[39] Wang CH, Wang LK, Wu CC, Chen ML, Lee MC, Lin YY (2019). The ribosomal protein RPLP0 mediates PLAAT4-induced cell cycle arrest and cell apoptosis. Cell Biochem Biophys.

[40] Chang TW, Chen CC, Chen KY, Su JH, Chang JH, Chang MC (2008). Ribosomal phosphoprotein P0 interacts with GCIP and overexpression of P0 is associated with cellular proliferation in breast and liver carcinoma cells. Oncogene..

[41] Artero-Castro A, Perez-Alea M, Feliciano A, Leal JA, Genestar M, Castellvi J (2015). Disruption of the ribosomal P complex leads to stress-induced autophagy. Autophagy..

[42] Blackford AN, Jackson SP (2017). ATM, ATR, and DNA-PK: The trinity at the heart of the DNA damage response. Mol Cell.

[43] Liccardi G, Hartley JA, Hochhauser D (2011). EGFR nuclear translocation modulates DNA repair following cisplatin and ionizing radiation treatment. Cancer Res.

[44] Yin XK, Wang YL, Wang F, Feng WX, Bai SM, Zhao WW (2021). PRMT1 enhances oncogenic arginine methylation of NONO in colorectal cancer. Oncogene..

[45] Wang YL, Liu JY, Yang JE, Yu XM, Chen ZL, Chen YJ (2019). Lnc-UCID promotes G1/S transition and hepatoma growth by preventing DHX9-mediated CDK6 down-regulation. Hepatology.

[46] Mace AG, Pai RK, Stocchi L, Kalady MF (2015). American Joint Committee on Cancer and College of American Pathologists regression grade: A new prognostic factor in rectal cancer. Dis Colon Rectum.

