# Supplementary material for: DNA damage-induced paraspeckle formation enhances DNA repair and tumor radioresistance by recruiting ribosomal protein P0
Source: Cell Death Dis. 2022 Aug 16;13(8):709. doi: 10.1038/s41419-022-05092-1 (PMC9381602; doi:10.1038/s41419-022-05092-1)
Supplement: Supplementary file 1 — Supplemental figures and table [file 41419_2022_5092_MOESM1_ESM.docx]

**Supplementary Figures**

**
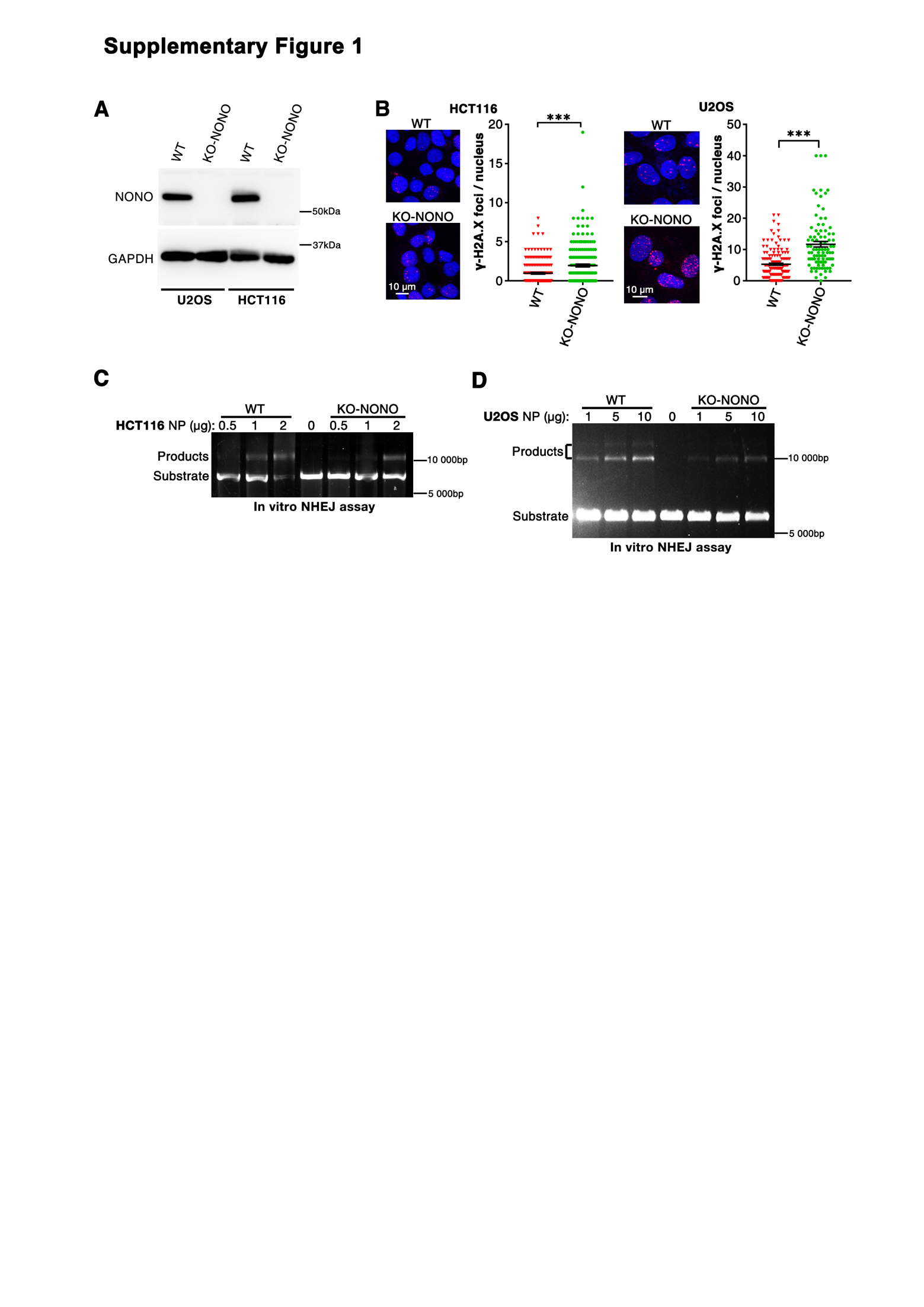
**

**Figure S1. NONO promotes DSB repair. (A)** Knocking out of NONO in HCT116 and U2OS cells. **(B)** Depletion of NONO significantly increased γ-H2A.X foci in tumor cells. Twenty hours after irradiation (2Gy), HCT116 and U2OS cells were fixed and subjected to immunofluorescence analysis. *n* = 201 (HCT116-WT), *n* = 197 (HCT116-KO); *n* = 158 (U2OS-WT), *n* = 93 (U2OS-KO). Scale bars, 10μm. **(C-D)** Knockout of NONO suppressed ligation of linearized DNA in NHEJ assay. pCSCMV-tdTomato plasmid was linearized with BamHI and incubated with nuclear proteins obtained from HCT116 (C) and U2OS (D) cells. The ligation product was separated using 0.8% agarose gels. ***, P < 0.001.


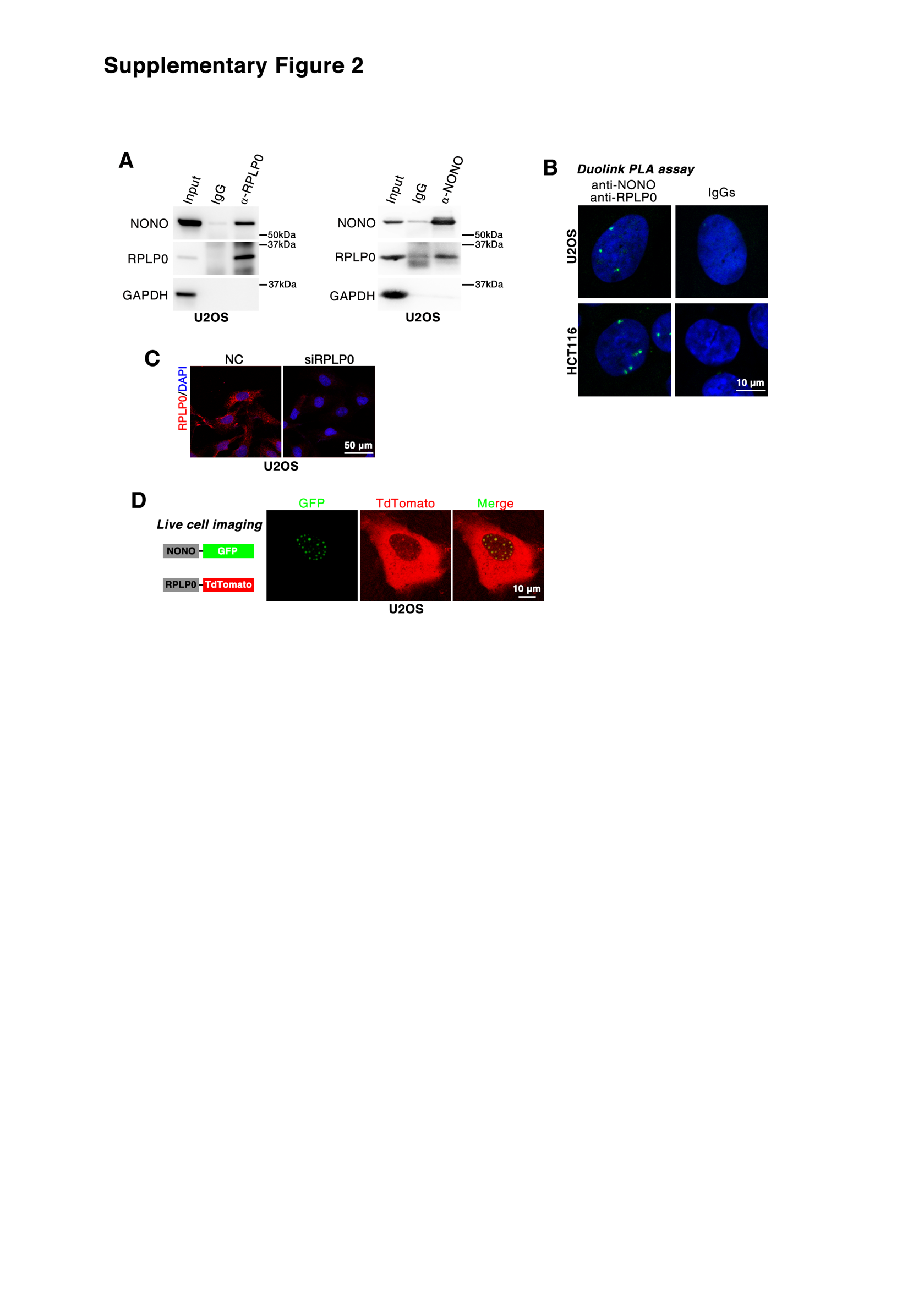


**Figure S2. Ribosomal protein RPLP0 interacts with NONO in nucleus. (A)** RPLP0 and NONO were associated in U2OS cells. NONO or RPLP0-binding protein was assessed by CoIP assay and analyzed with Western blotting. **(B)** The association of NONO and RPLP0 was examined with Duolink PLA assay in cell lines. Scale bar, 10μm. **(C)** Verify the specifity of anti-RPLP0 antibody using siRPLP0. U2OS cells were transfected with siRNA for 48h before IF assay. Scale bars, 50μm. **(D)** RPLP0-tdTomato and NONO-GFP colocalize in U2OS cells. Twenty-four hours after transfection with RPLP0-tdTomato and NONO-GFP plasmids, U2OS live cells were analyzed using confocal microscopy. Scale bars, 10μm.

**
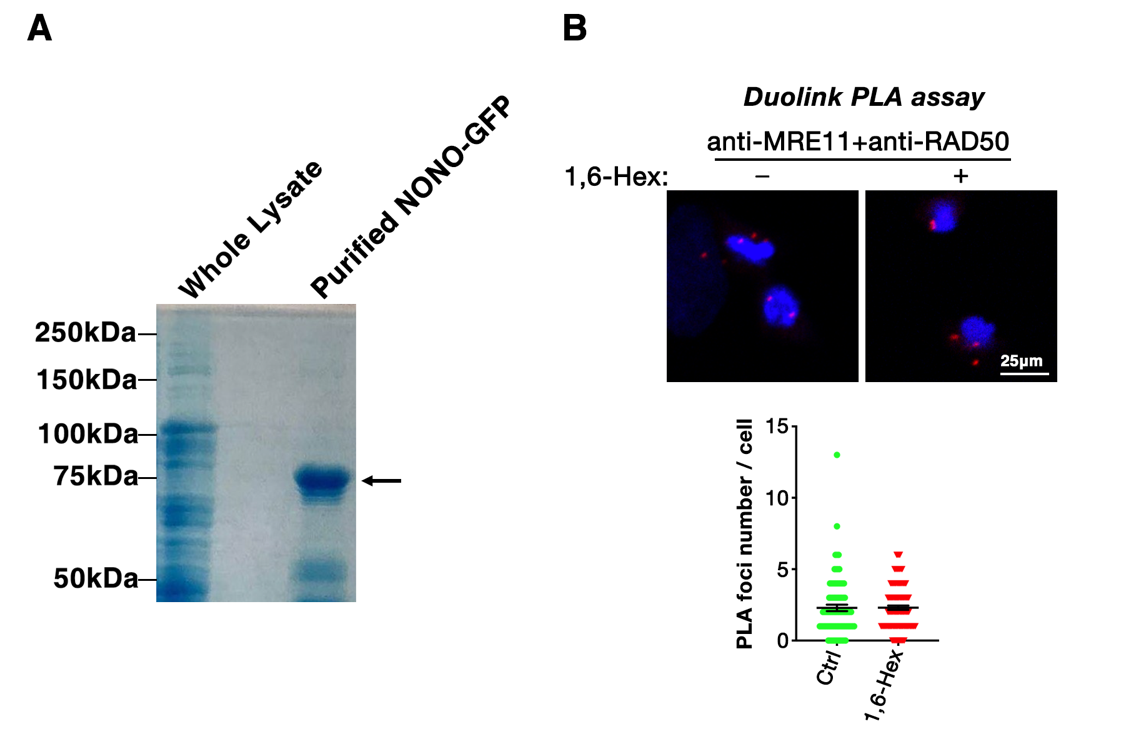
**

**Figure S3. (A)** The purified NONO-GFP protein was separated with SDS-PAGE. **(B)** 1,6-hexanediol has no impact on the interaction of MRE11 and RAD50. *n* = 84 (Ctrl), *n* = 111 (1,6-Hex). Scale bar, 25μm.

**
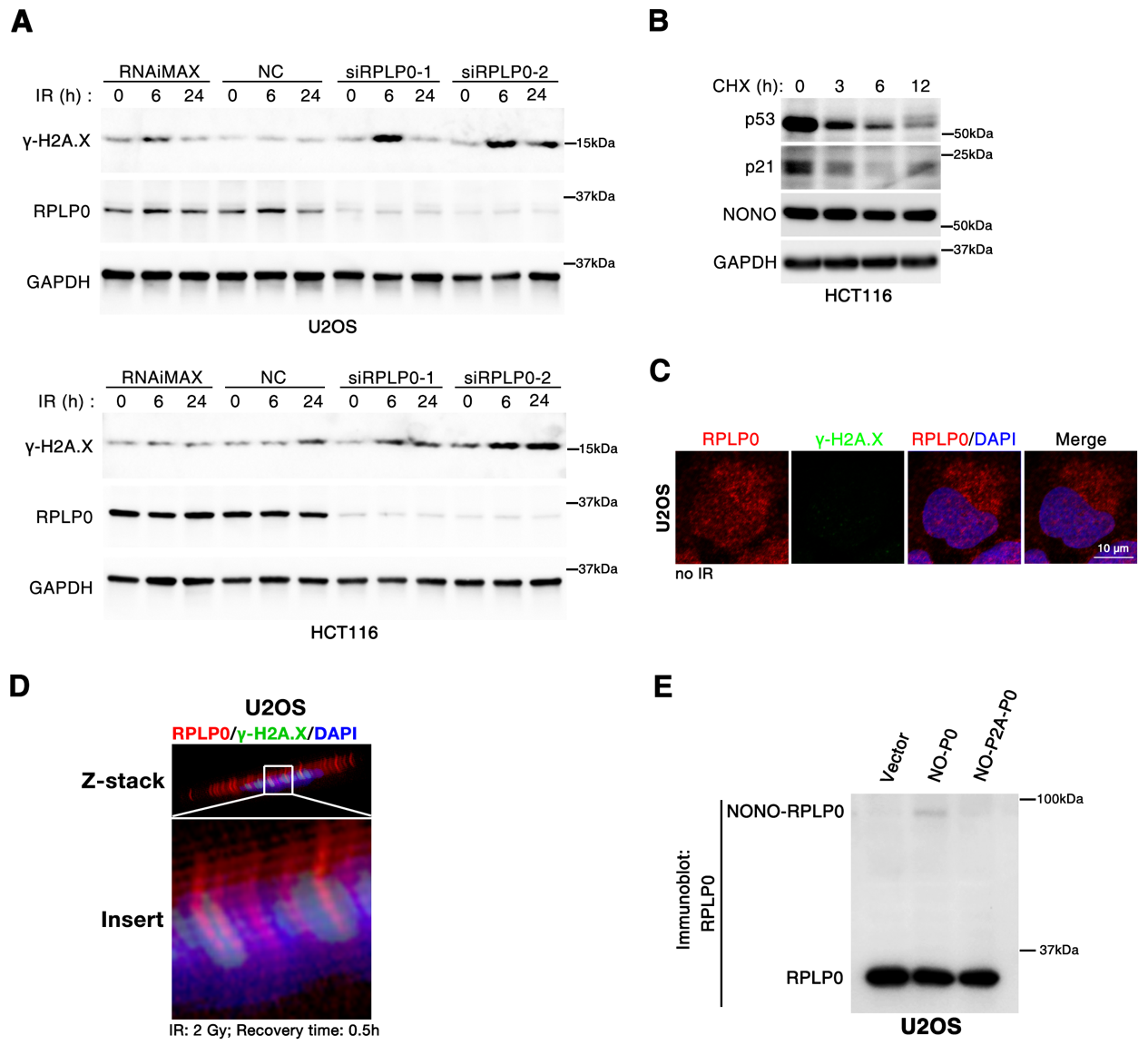
**

**Figure S4. (A)** siRPLP0 increased the γ-H2A.X level at both 6 and 24 hours after IR. (**B**) CHX treatment reduced the protein level of p53 and p21, whereas has no influence on NONO expression. **(C)** IF assay were performed to verify the colocalization of RPLP0 and γ-H2A.X. Scale bars, 10μm. **(D)** Z-stack imaging confirmed the colocalization of RPLP0 and γ-H2A.X in the nucleus. **(E)** The expression of NONO-RPLP0, NONO-P2A-RPLP0 fusion protein was analyzed by Western blotting.

**
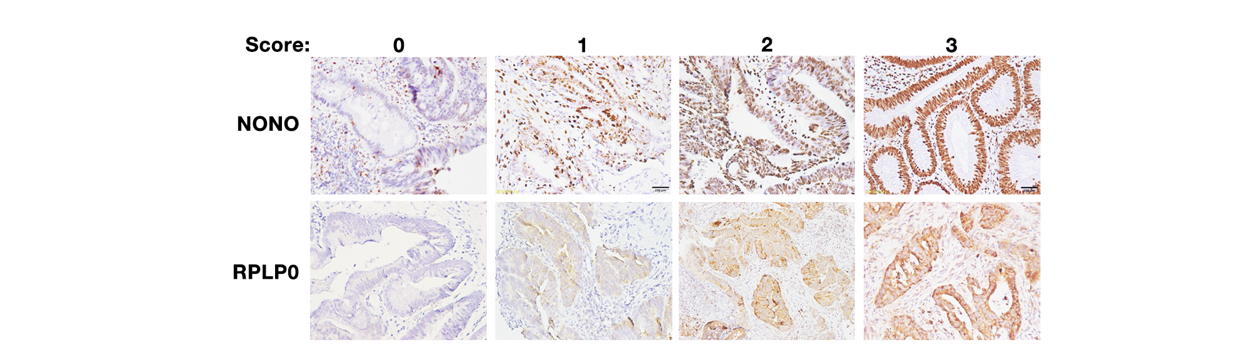
**

**Figure S5. The representative images of NONO and RPLP0 IHC assay in rectal cancer tissues.**

**
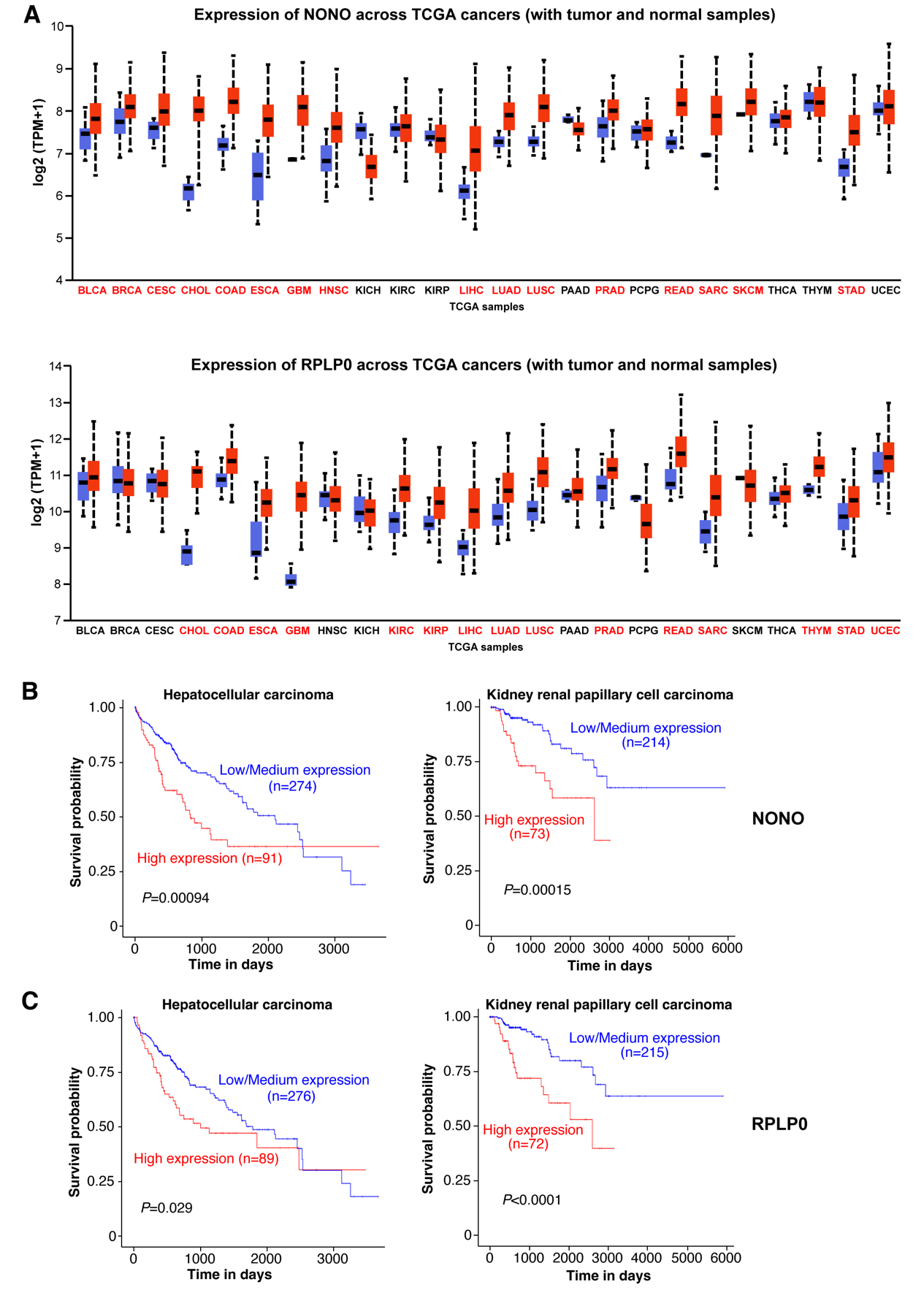
**

**Figure S6. RPLP0 was dysregulated in different types of cancer. (A)** NONO and RPLP0 were upregulated in varies types of cancer. BLCA, Bladder Urothelial Carcinoma; BRCA, Breast Invasive Carcinoma; CESC, Cervical squamous cell carcinoma; CHOL, Cholangiocarcinoma; COAD, Colon Adenocarcinoma; ESCA, Esophageal Carcinoma; GBM, Glioblastoma multiforme; HNSC, Head and Neck Squamous Cell Carcinoma; KICH, Kidney Chromophobe; KIRC, Kidney Renal Clear Cell Carcinoma; KIRP, Kidney Renal Papillary; LIHC, Liver hepatocellular carcinoma; LUAD, Lung Adenocarcinoma; LUSC, Lung Squamous Cell Carcinoma; PAAD, Pancreatic Adenocarcinoma; PRAD, Prostate Adenocarcinoma; PCPG, Pheochromocytoma and Paraganglioma; READ, Rectum adenocarcinoma; SARC, Sarcoma; SKCM, Skin Cutaneous Melanoma; STAD, Stomach Adenocarcinoma; THYM, Thymoma; THCA, Thyroid Carcinoma; UCEC, Uterine Corpus Endometrial Carcinoma. **(B-C)** The higher expression of NONO and RPLP0 were correlated with poorer survival of cancer patients. For (A-C), The Cancer Genome Atlas (TCGA) data were analyzed using the UALCAN webtool (<http://ualcan.path.uab.edu/index.html>).

**Supplementary Table S1. Sequences of DNA and RNA oligonucleotides.**

| **Name** | **Sense Strand/Sense Primer (5' - 3')** | **Antisense Strand/Antisense Primer (5' - 3')** |
| --- | --- | --- |
| **siRNA duplexes** | |  |
| siRPLP0-1 | GAUCAAGACUGGAGACAAAdTdT | UUUGUCUCCAGUCUUGAUCdAdG |
| siRPLP0-2 | CCAAGGAAGAGUCGGAGGAdTdT | UCCUCCGACUCUUCCUUGGdCdT |
| siNEAT1-1 | CAGGAGGCUACCAUUUAAATT | UUUAAAUGGUAGCCUCCUGTT |
| siNEAT1-2 | GCCUUGUAAAUGCCUAUAUTT | AUAUAGGCAUUUACAAGGCTT |
| NC | UUCUCCGAACGUGUCACGUdTdT | ACGUGACACGUUCGGAGAAdTdT |
| **Primers for qPCR** | | |
| NONO | TATGGAAAGGCAGGCGAAGT | TGGCATATTGTCCAGCTCCA |
| RPLP0 | TGGCAGCATCTACAACCCTG | GACAAGGCCAGGACTCGTTT |
| NEAT1 | GTGGCTGTTGGAGTCGGTAT | TAACAAACCACGGTCCATGA |
| 28s rRNA | TCATCAGACCCCAGAAAAGG | GATTCGGCAGGTGAGTTGTT |
| GAPDH | GAGTCAACGGATTTGGTCGT | GACAAGCTTCCCGTTCTCAG |
| U6 | CGGCAGCACATATAC | TTCACGAATTTGCGTGTCAT |
|  |  |  |
| **Primers for ChIP** | | |
| F+R | GGGTATGGAATTTGGCCCCA | CAACCCGATCCCTCCACTTG |
