# Supplementary material for: DNA damage-induced paraspeckle formation enhances DNA repair and tumor radioresistance by recruiting ribosomal protein P0
Source: Cell Death Dis. 2022 Aug 16;13(8):709. doi: 10.1038/s41419-022-05092-1 (PMC9381602; doi:10.1038/s41419-022-05092-1)

Figure 1

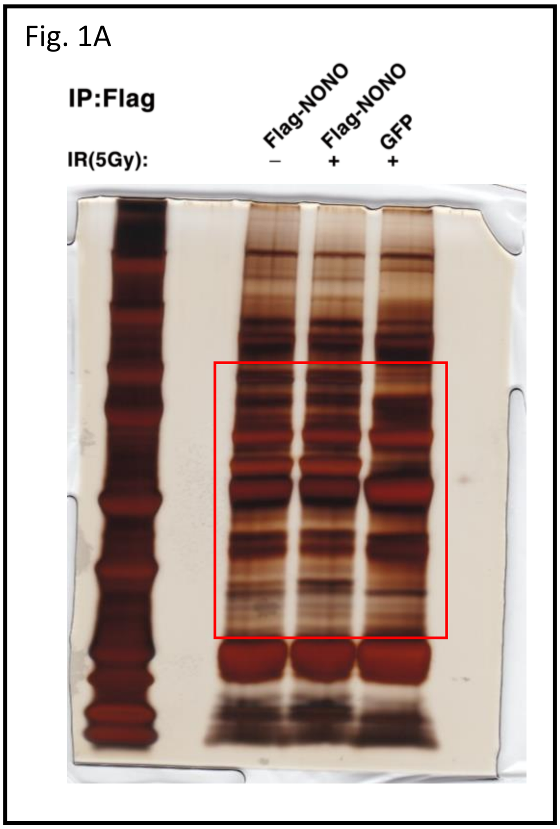
Fig. 1A
IP:Flag
IR(5Gy):
Flag-NONO
Flag-NONO
GFP
–
+
+

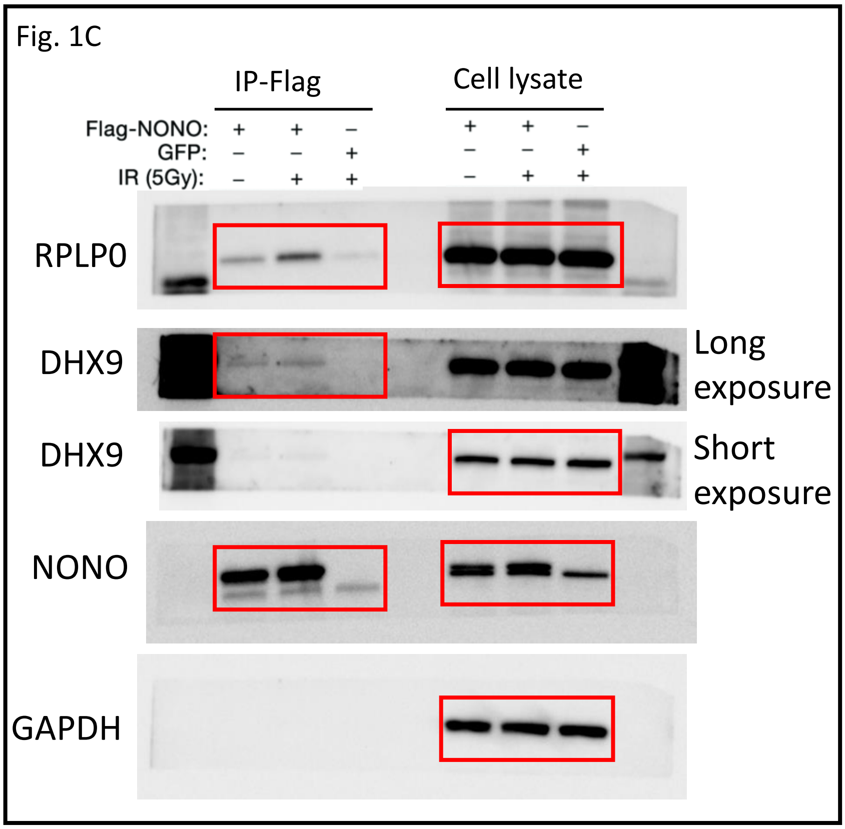
Fig. 1C
IP-Flag
Cell lysate
Flag-NONO: + + – + + –
GFP: – – + – – +
IR (5Gy): – + + – + +
RPLP0
DHX9
Long exposure
DHX9
Short exposure
NONO
GAPDH

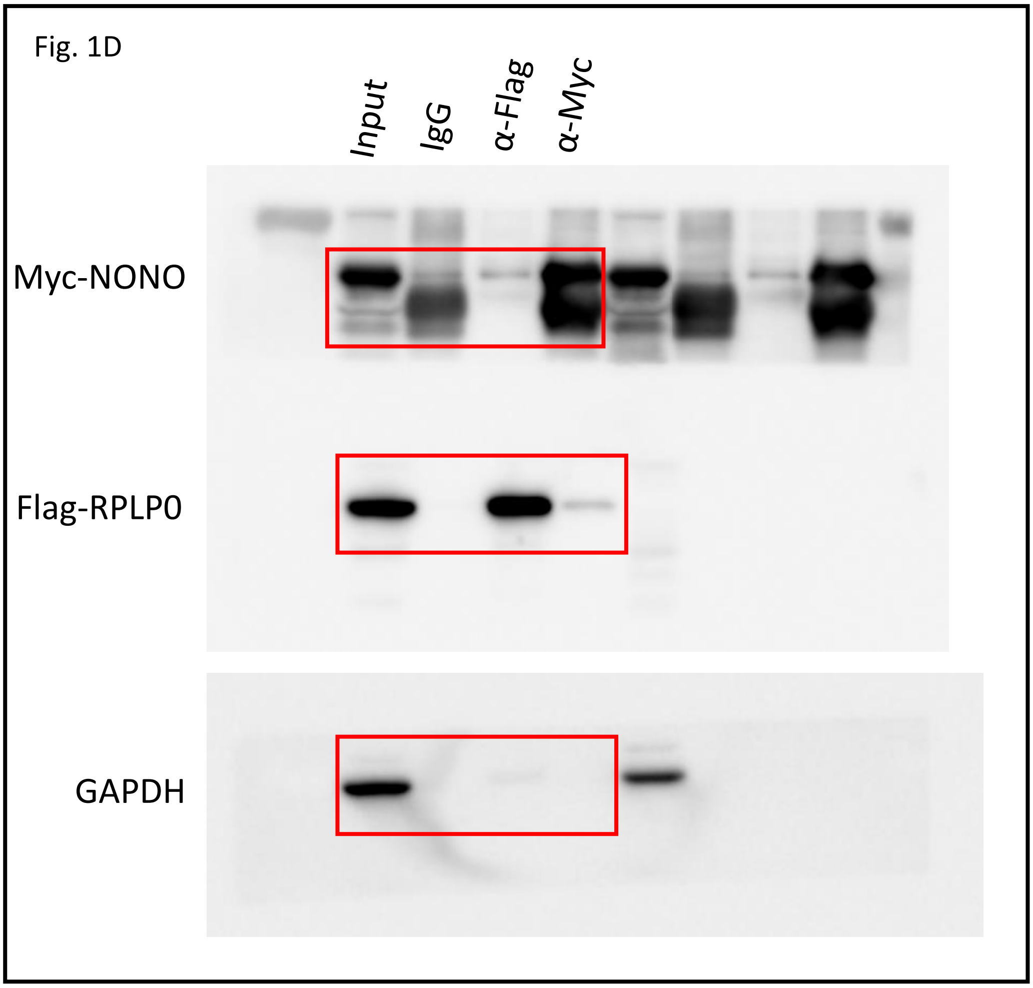
Fig. 1D
Input
IgG
α-Flag
α-Myc
Myc-NONO
Flag-RPLP0
GAPDH

Figure 1

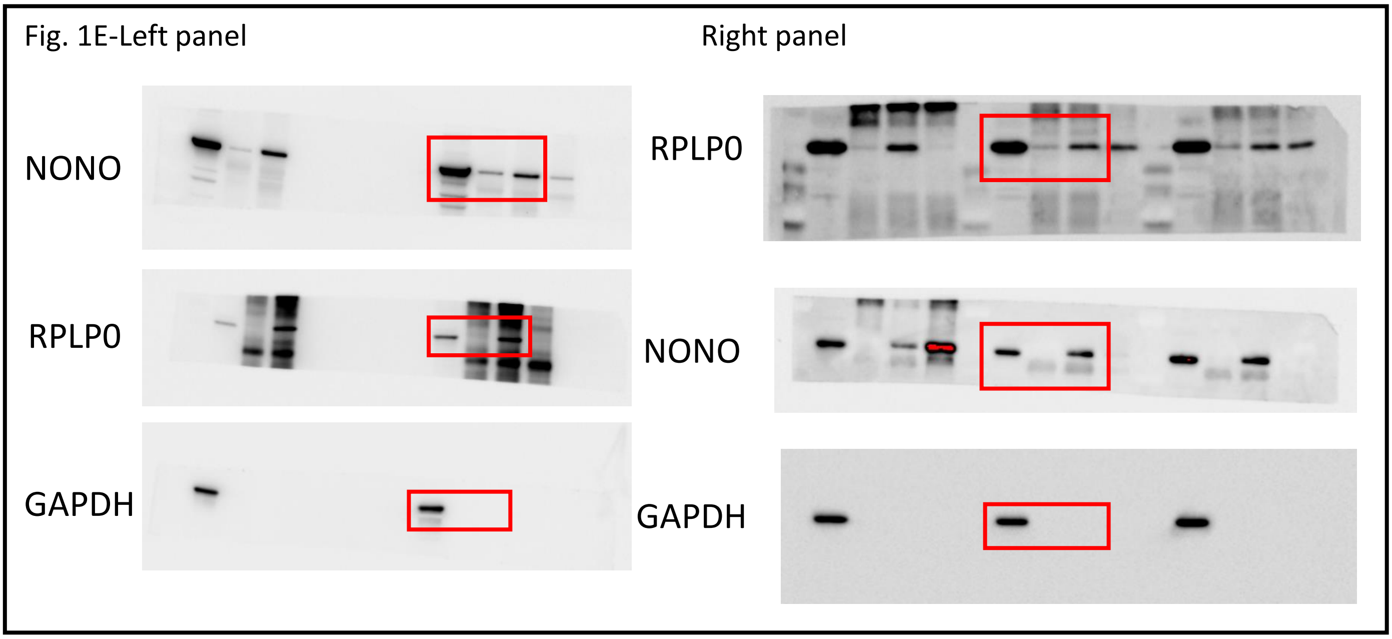
Fig. 1E-Left panel
NONO
RPLP0
GAPDH
Right panel
RPLP0
NONO
GAPDH

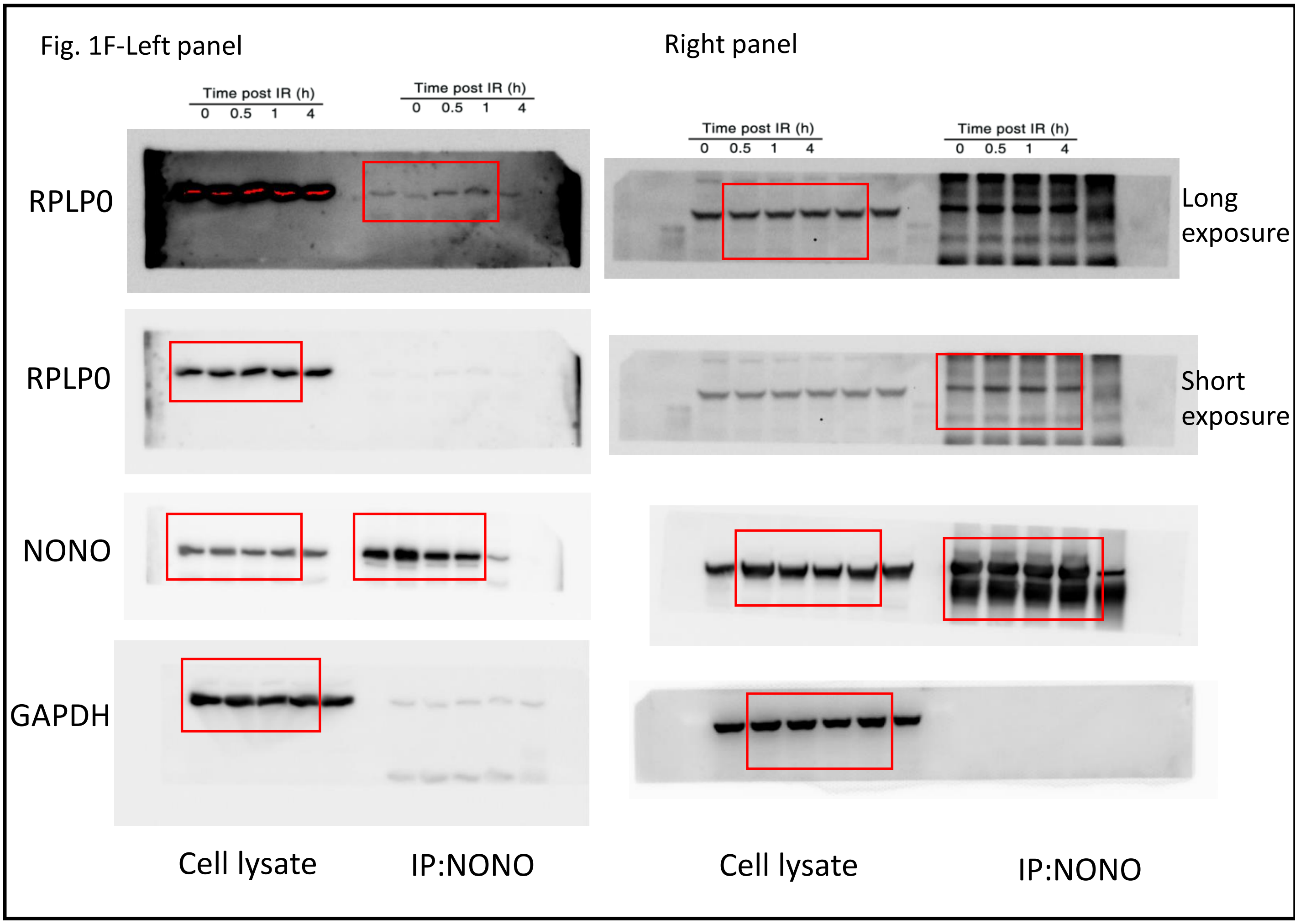
Fig. 1F-Left panel
Time post IR (h)
0 0.5 1 4
Time post IR (h)
0 0.5 1 4
RPLP0
RPLP0
NONO
GAPDH
Cell lysate
IP:NONO
Right panel
Time post IR (h)
0 0.5 1 4
Time post IR (h)
0 0.5 1 4
Long exposure
Short exposure
Cell lysate
IP:NONO

Figure 2

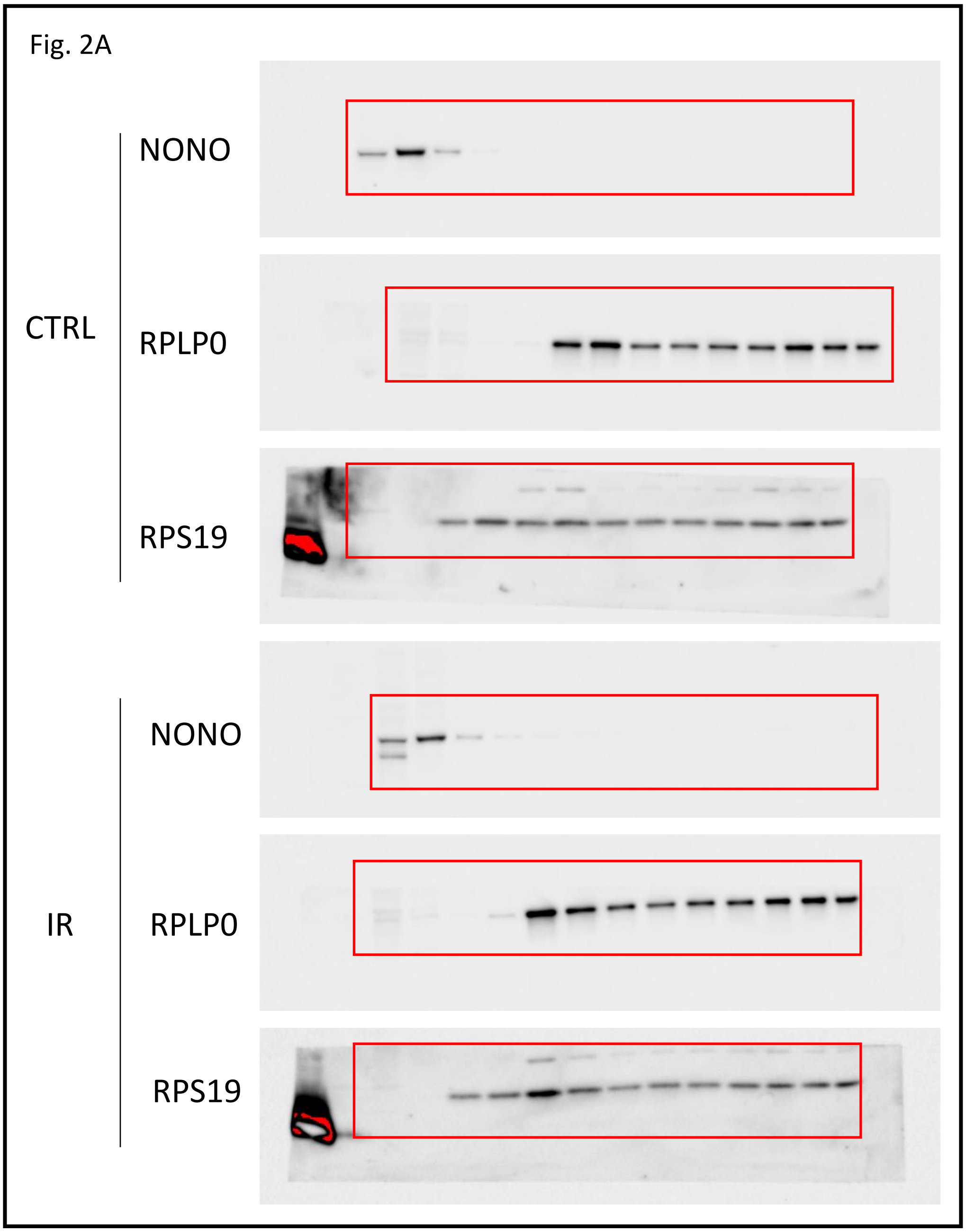

Fig. 2A
CTRL
NONO
RPLP0
RPS19
IR
NONO
RPLP0
RPS19

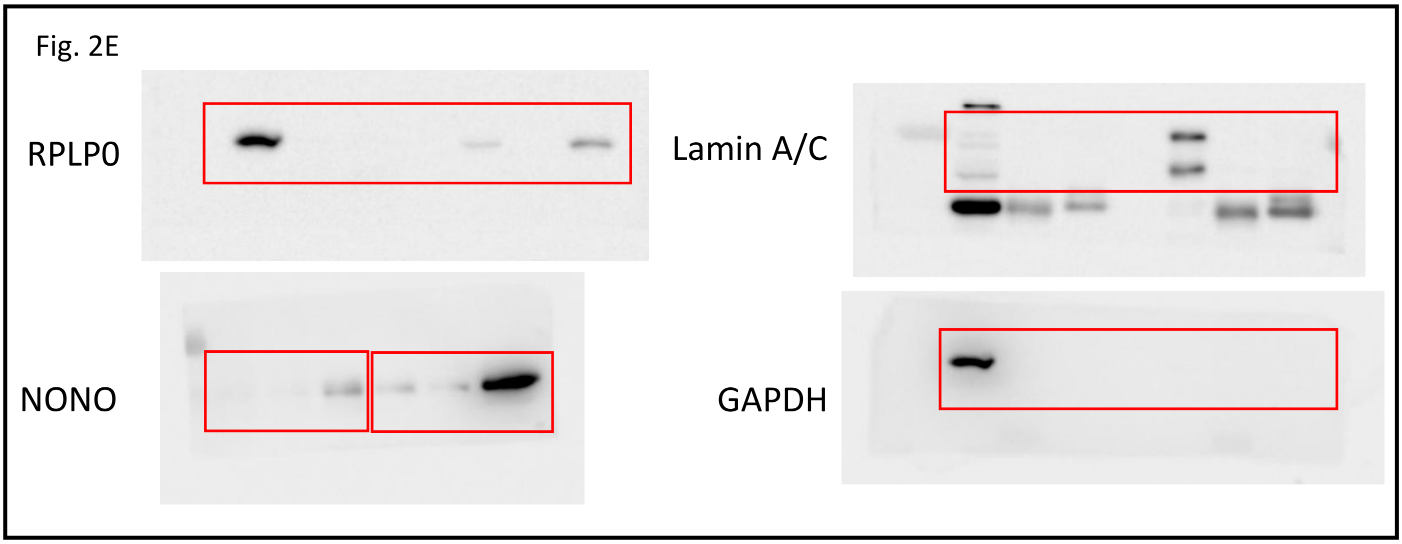

Fig. 2E
RPLP0
Lamin A/C
NONO
GAPDH

Figure 2

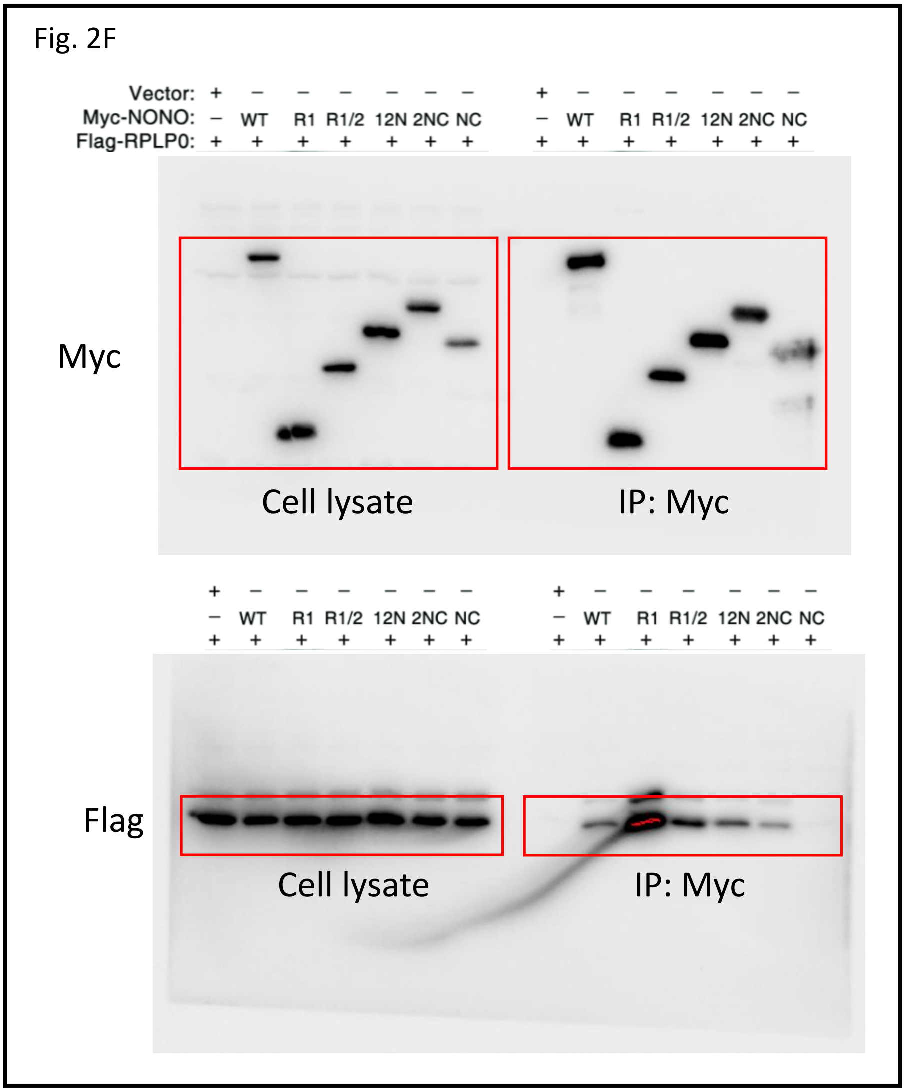

Fig. 2F
Vector: + – – – – – – + – – – – – –
Myc-NONO: – WT R1 R1/2 12N 2NC NC – WT R1 R1/2 12N 2NC NC
Flag-RPLP0: + + + + + + + + + + + + + +
Myc
Cell lysate
IP: Myc
Flag
Cell lysate
IP: Myc

Figure 3

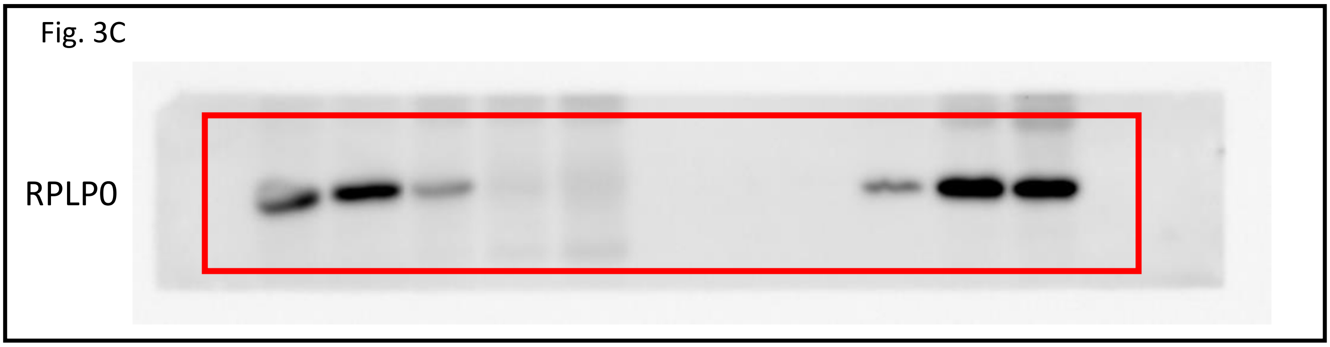
Fig. 3C
RPLPO

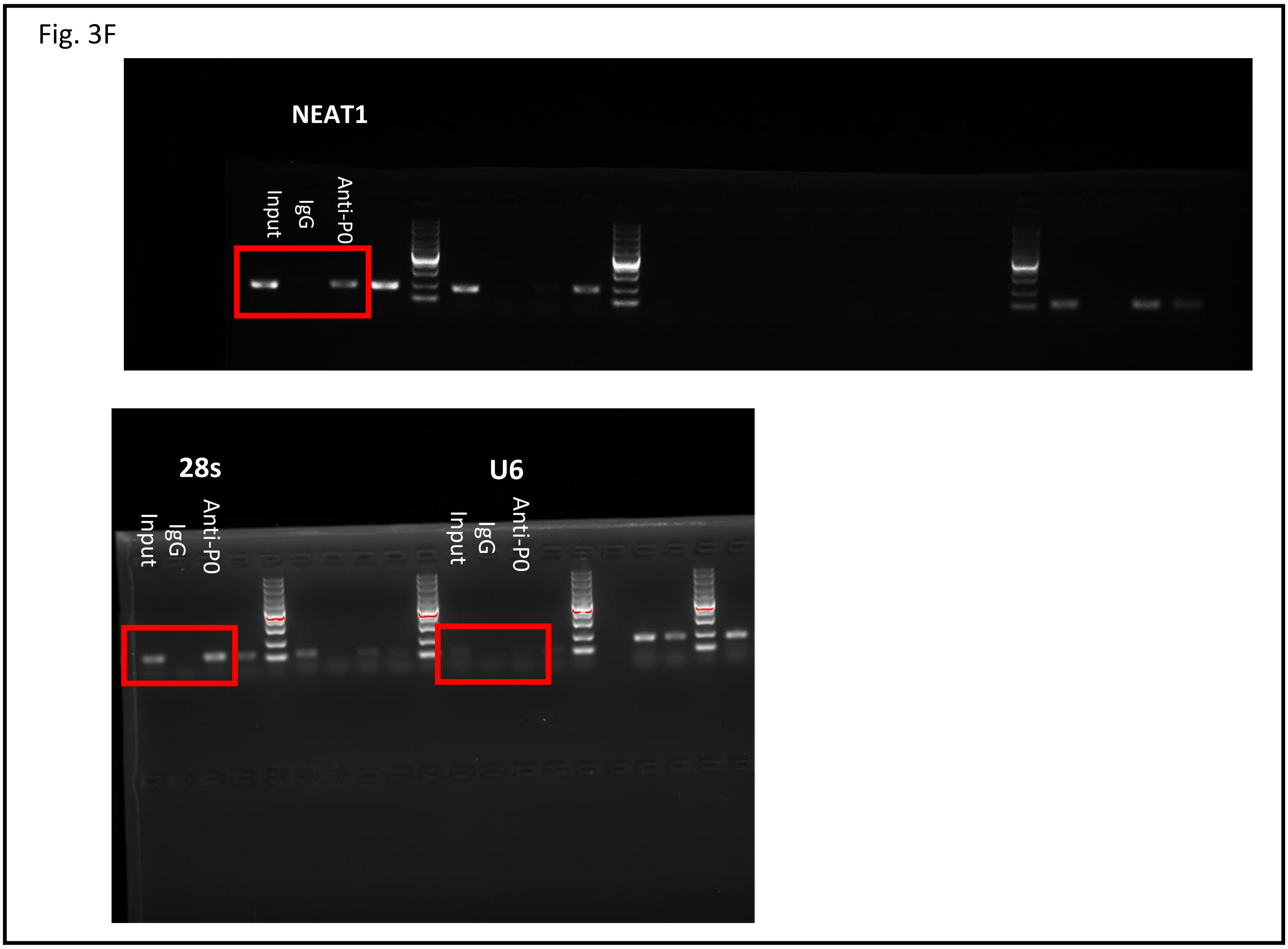
Fig. 3F
NEAT1
Input
IgG
Anti-P0
28s
Input
IgG
Anti-P0
U6
Input
IgG
Anti-P0

Figure 3

Fig. 3G

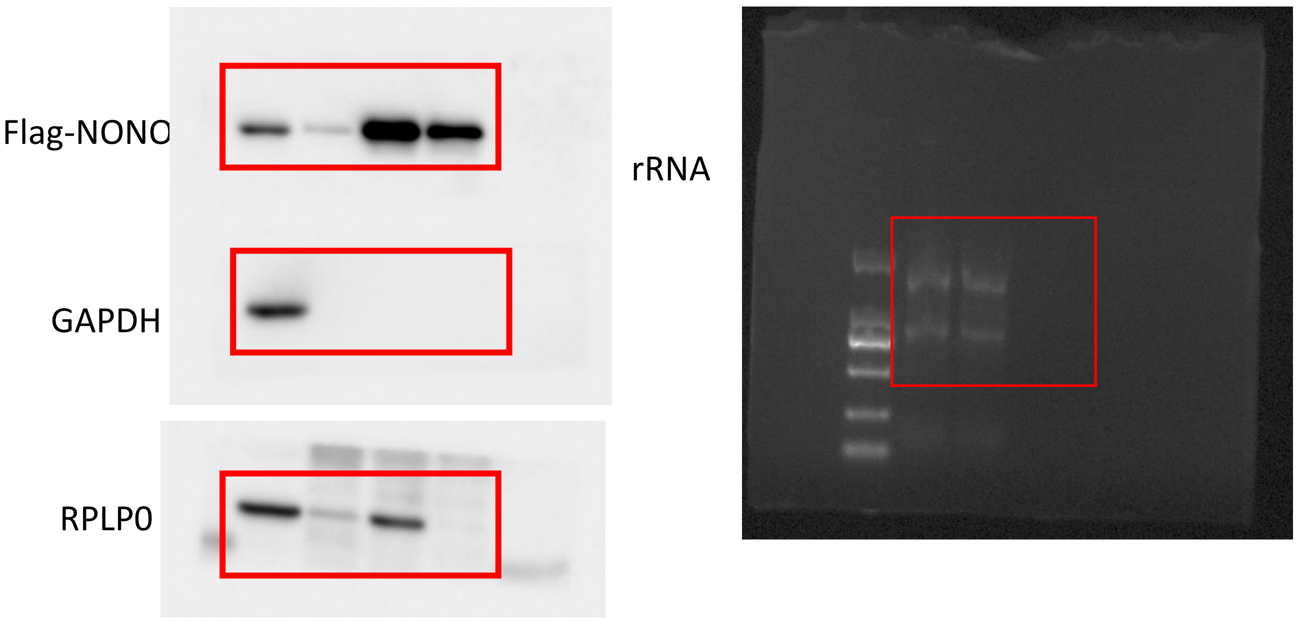

Fig. 3H-Left panel

Right panel

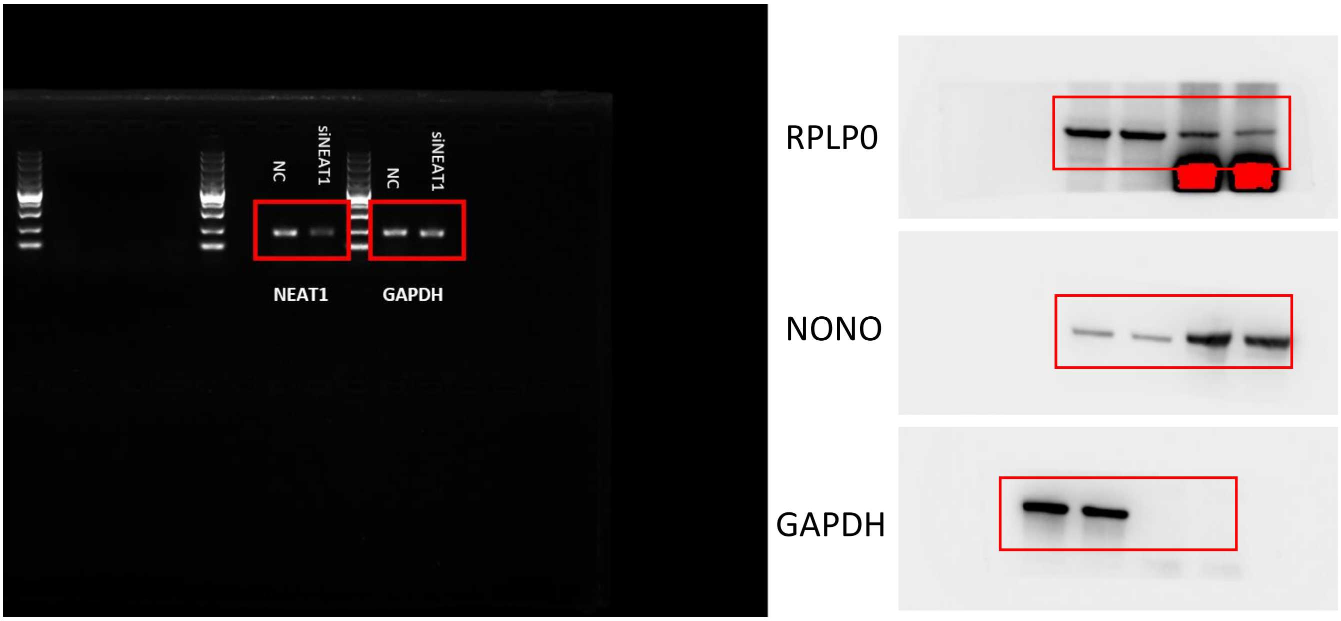

Figure 4

Fig. 4A-Top panel

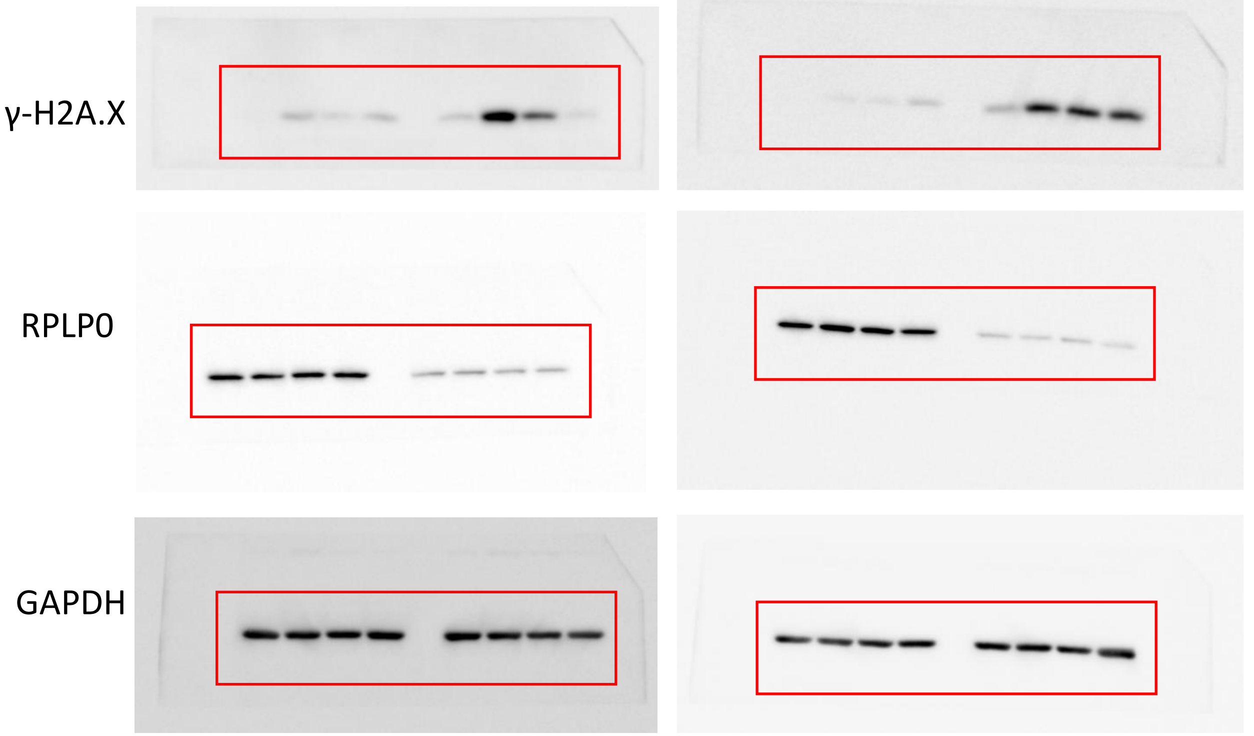

Fig. 4A-Bottom panel

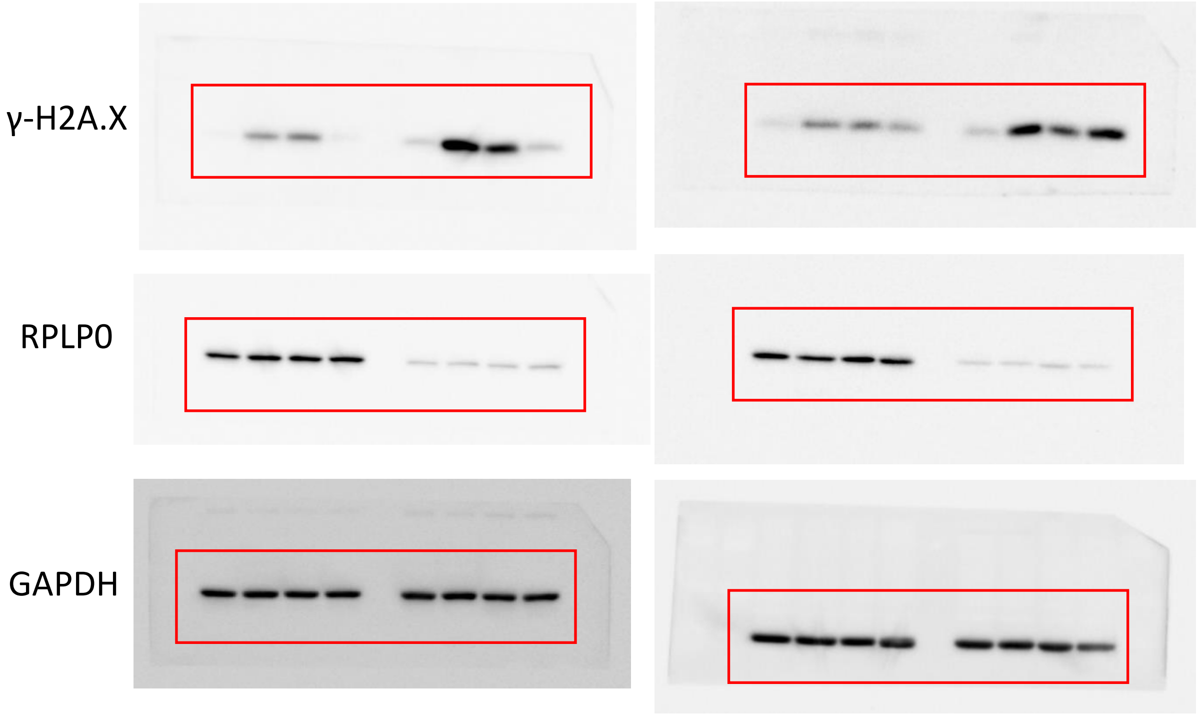

Figure 4

Fig. 4E

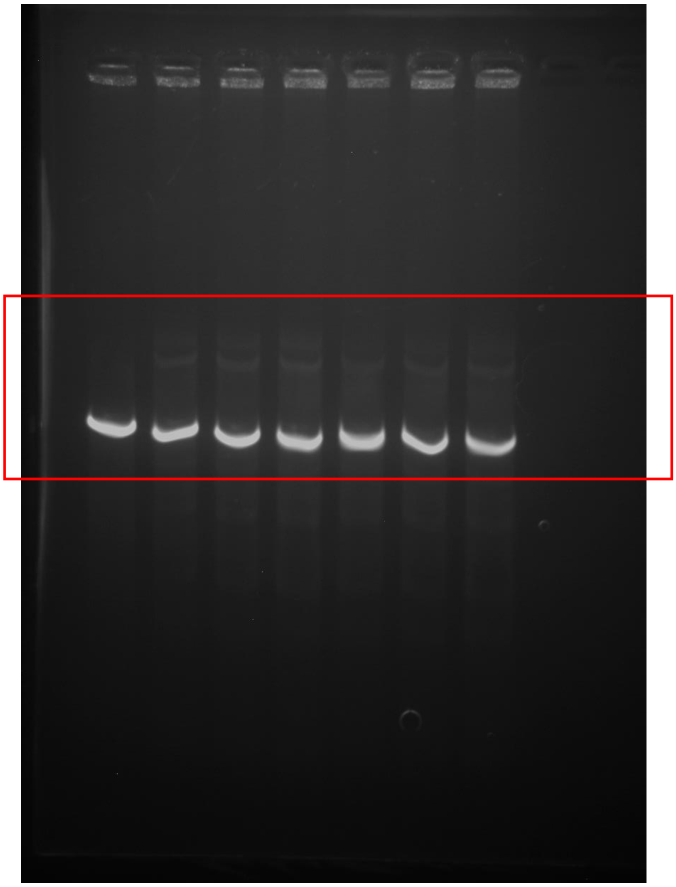

Fig. 4G

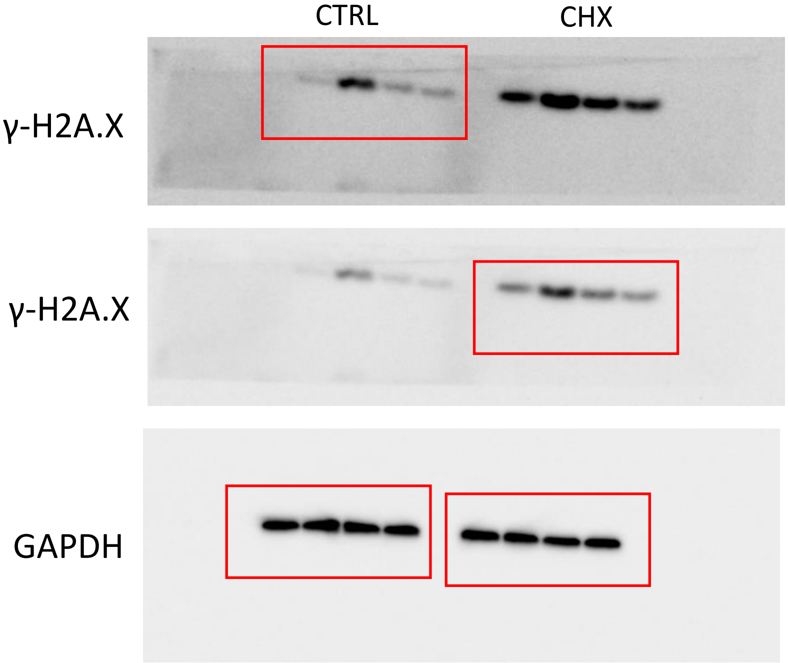

Figure 5

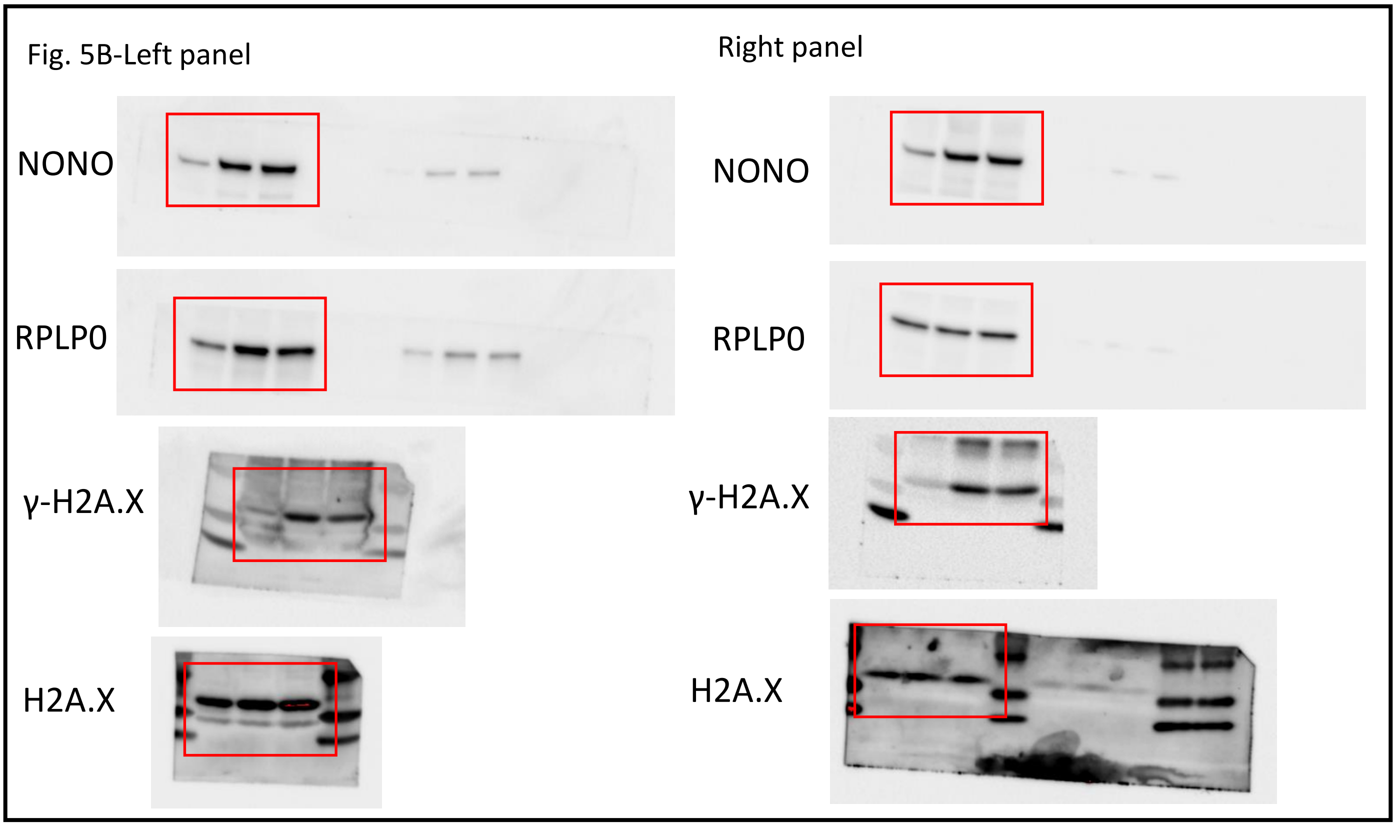
Fig. 5B-Left panel
Right panel
NONO
RPLPO
γ-H2A.X
H2A.X
NONO
RPLPO
γ-H2A.X
H2A.X

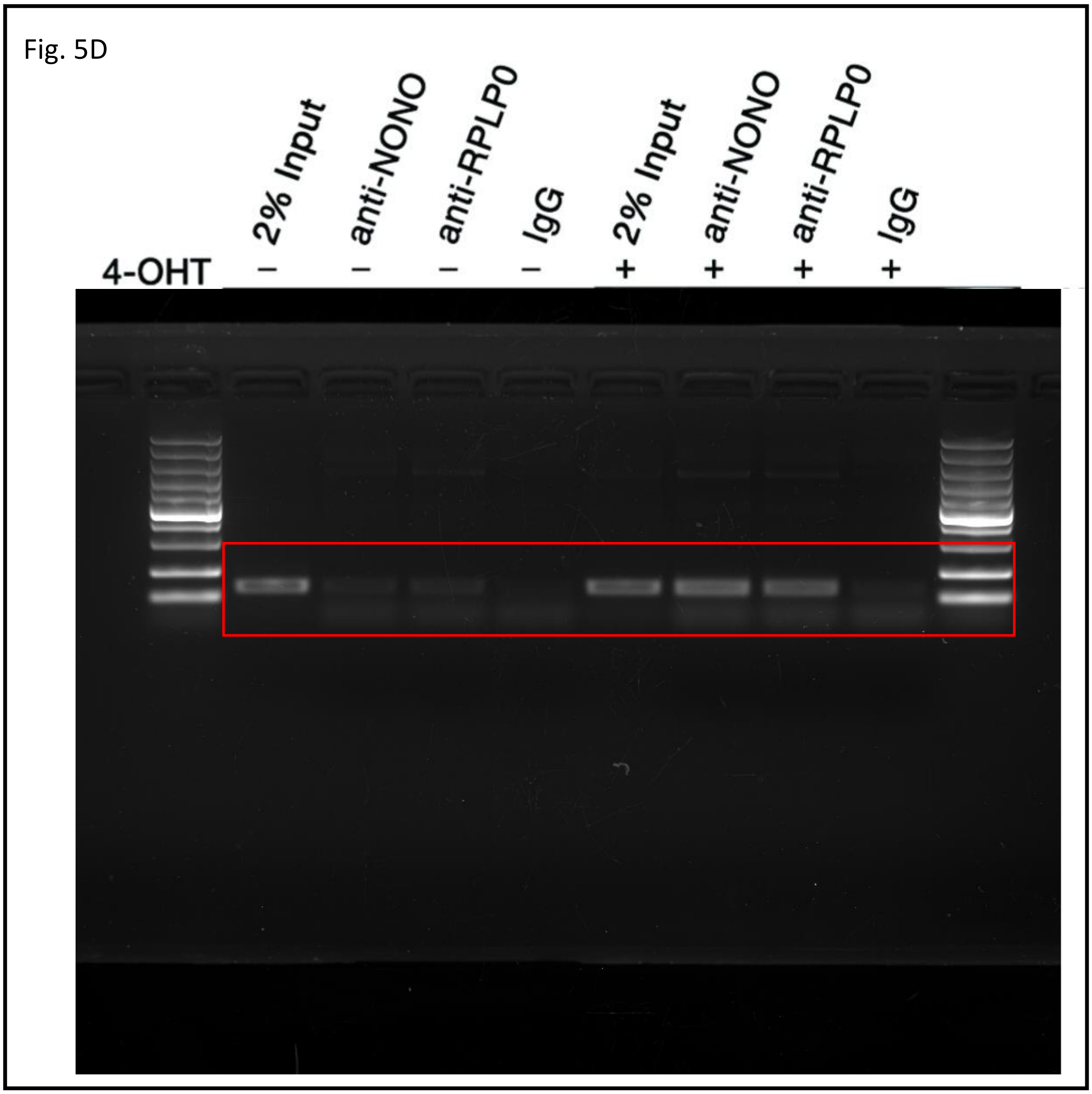
Fig. 5D
4-OHT
2% Input
anti-NONO
anti-RPLPO
IgG
2% Input
anti-NONO
anti-RPLPO
IgG
– – – – + + + +

Figure 5

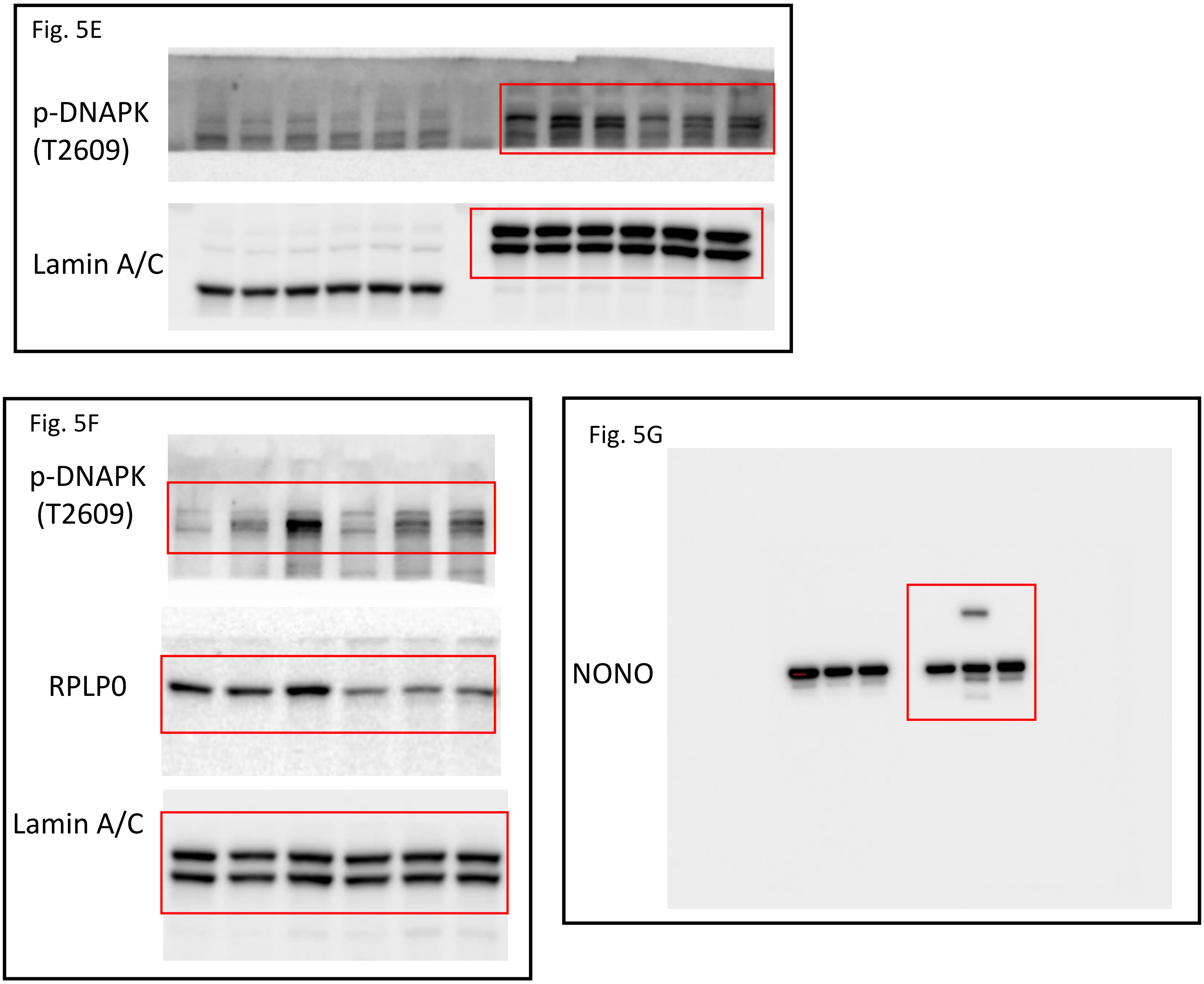

Fig. 5E
p-DNAPK (T2609)
Lamin A/C
Fig. 5F
p-DNAPK (T2609)
RPLP0
Lamin A/C
Fig. 5G
NONO

Figure 6

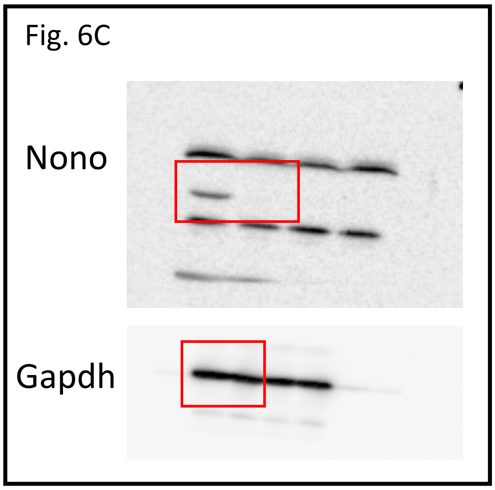

Fig. 6C
Nono
Gapdh

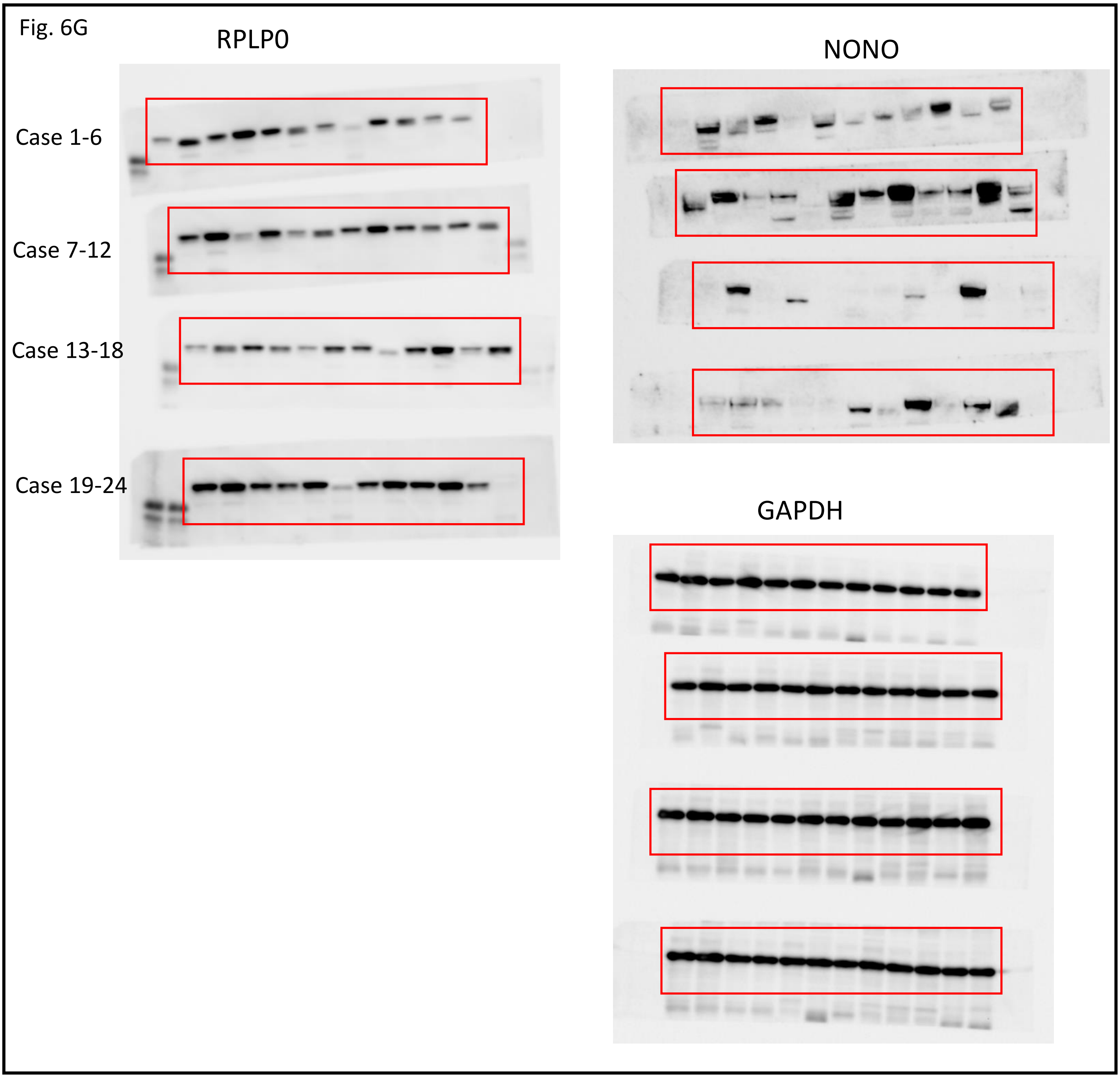

Fig. 6G
RPLPO
NONO
Case 1-6
Case 7-12
Case 13-18
Case 19-24
GAPDH

Supplementary Figure 1

Fig. S1A

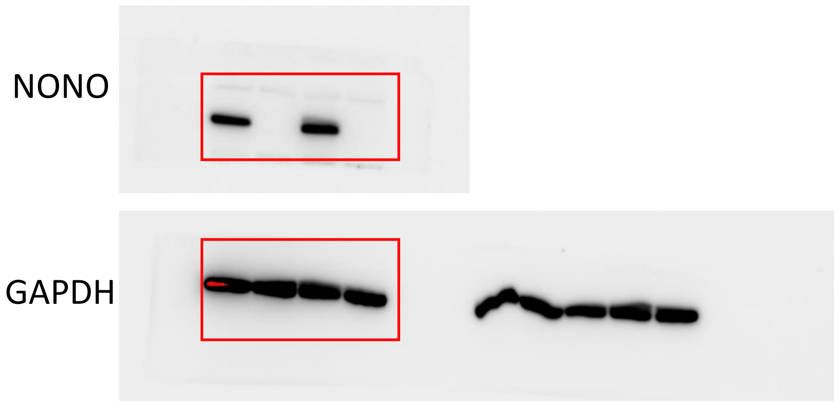

Fig. S1C

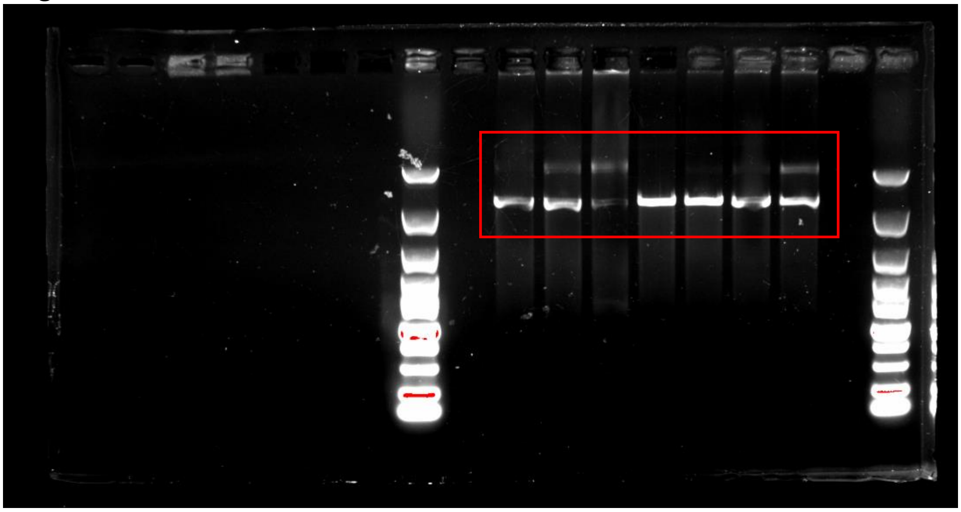

Fig. S1D

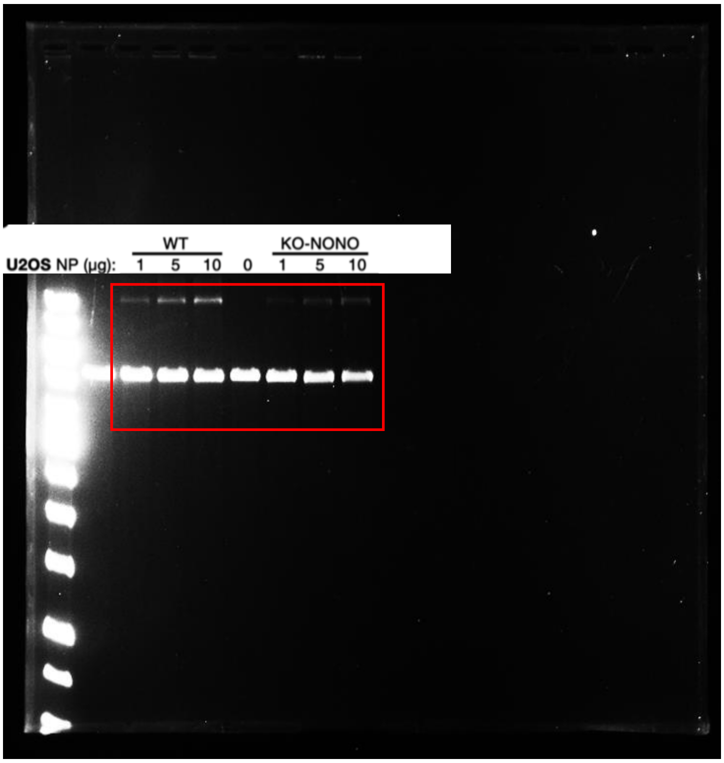

Supplementary Figure 2 and 3

Fig. S2A-Left panel Right panel

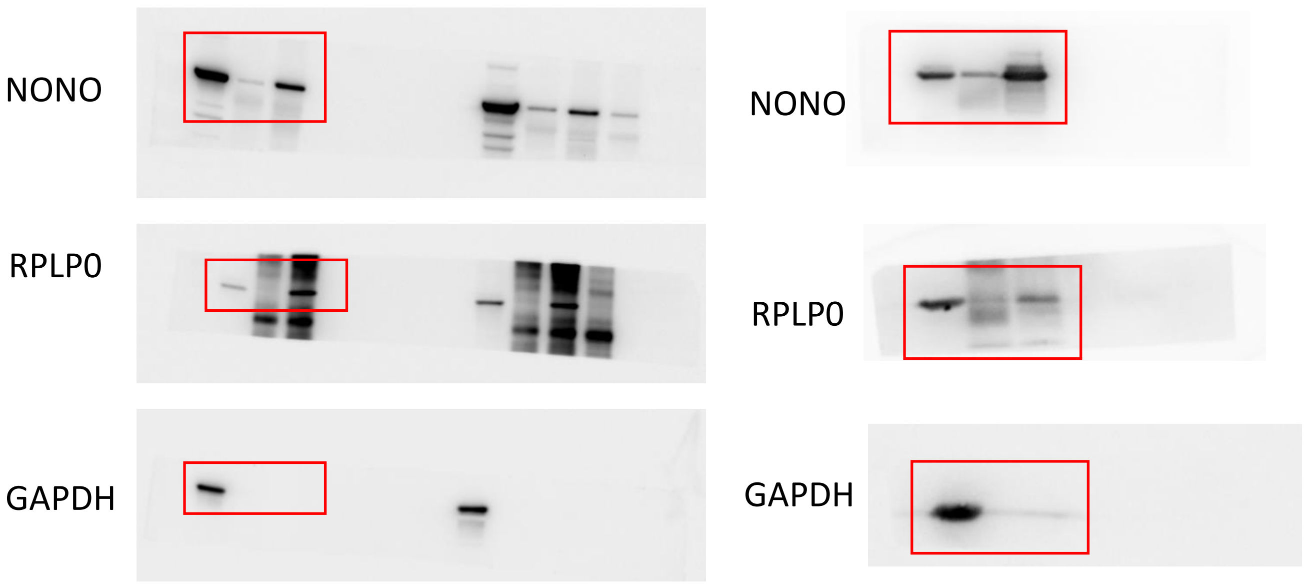

Fig. S3A

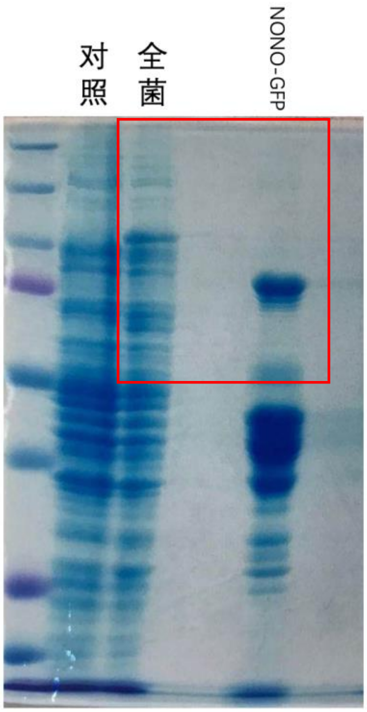

Supplementary Figure 4

Fig. S4A-Top panel

γ-H2A.X

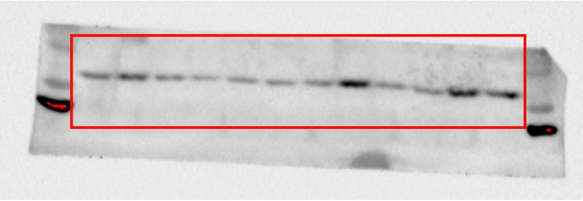

RPLP0

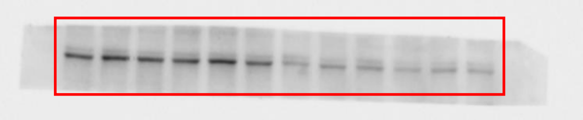

GAPDH

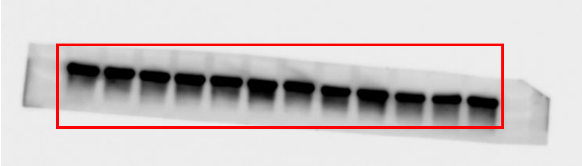

Bottom panel

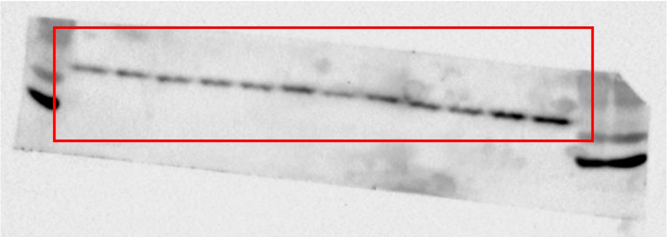

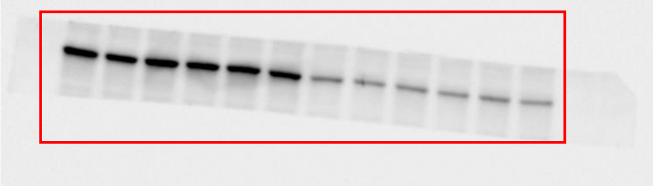

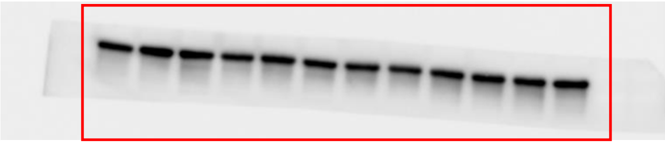

Fig. S4B

P53

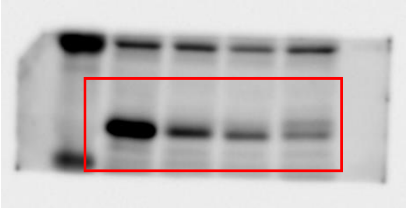

P21

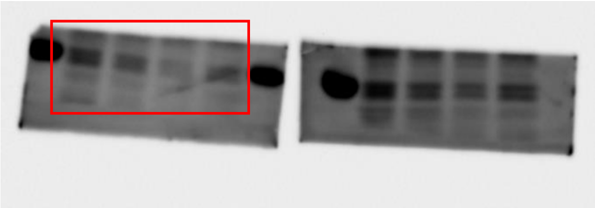

NONO

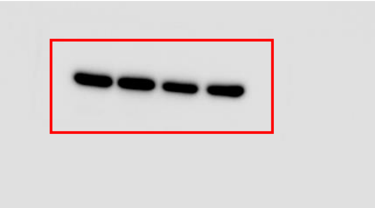

GAPDH

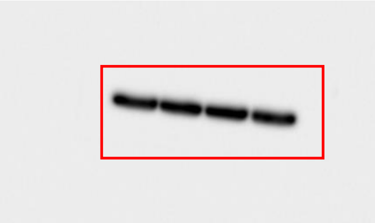

Fig. S4E

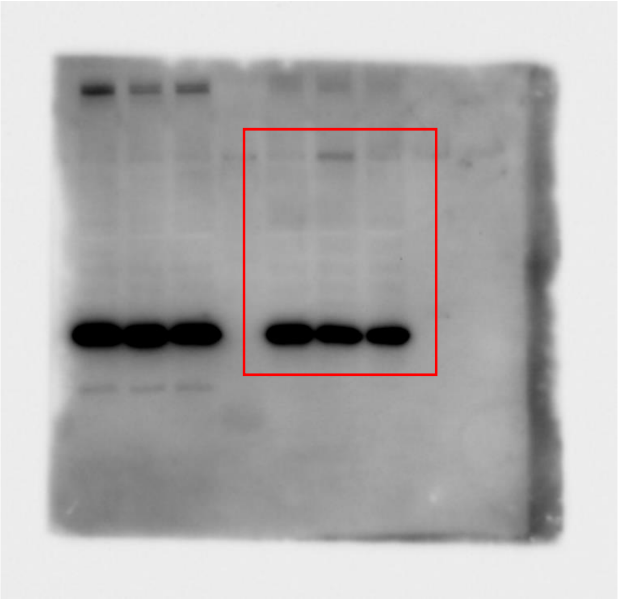

Supplement: Supplementary file 2 — Original western blots and DNA gels [file 41419_2022_5092_MOESM2_ESM.pdf]
